# Supplementary material for: Expanding CyanoHAB Monitoring: New Micropeptins and Generalizable MS/MS Workflows for the Annotation of Cyanopeptide Classes
Source: bioRxiv. 2026 Feb 9:2026.02.07.704577. Preprint. [Version 1] doi: 10.64898/2026.02.07.704577 (PMC12918887; doi:10.64898/2026.02.07.704577)
Supplement: Supplement 1 [file media-1.pdf]

Supporting Information: Expanding CyanoHAB Monitoring: New Micropeptins and Generalizable MS/MS Workflows for the Annotation of Cyanopeptide Classes

Runjie Xia,[a] Lindsey Ahn,[a] Michaela Burkhauser,[a] Ross Youngs,[b] and Matthew J. Bertin[a,]*

[a]Department of Chemistry, Case Western Reserve University, Cleveland, OH 44106, United States

[b]Biosortia, Inc., 2545 Farmers Dr., Suite 370, Columbus, OH 43235, United States

**Corresponding author. Email address: mxb1224@case.edu*

Additional Experimental Procedures

General Experimental Procedures. Optical rotation values were measured using a Jasco P-2000 polarimeter. NMR spectra were recorded on a Bruker 500 MHz Ascend Advance III NMR instrument. Chemical shifts reported for **1-3** were referenced to the residual solvent peak of $(CD_3)_2SO$ (δ_H 2.50 and δ_C 39.5). LC-HRMS and LC-HRMS^2 data were collected on an Agilent Revident QTOF mass spectrometer equipped with a Jet Stream source and 1290 Infinity II Bio LC (with multisampler and multicolumn thermostat) and MassHunter Workstation software. Additional LC-MS analyses were conducted on an Agilent LC-MSD single-quadrupole mass spectrometer equipped with an Agilent 1260 HPLC system with autosampler. Semipreparative HPLC separations were carried out using an Agilent 1260 Infinity system equipped with a vacuum degasser, autosampler, and diode array detector.

Configuration Analysis of 1-3. To determine the absolute configuration of the α-amino acids in compounds **1–3**, 0.4 mg of each compound was hydrolyzed in 0.5 mL of 6 N HCl at 110°C for 16 h. After cooling to room temperature, the hydrolysates were dried under a stream of nitrogen, reconstituted in 100 µL of water and 100 µL of 1 M $NaHCO_3$. Derivatization was performed by the addition of 500 µL of a 1% (w/v) solution of *N*-α-(2,4-dinitro-5-fluorophenyl)-L-valinamide (L-FDVA) in acetone. The reaction mixtures were stirred and heated at 40°C for 1 h, then quenched by the addition of 50 µL of 2 N HCl. The derivatized hydrolysates were subsequently diluted 1:10 with a 1:1 mixture of H_2O/CH_3CN to a final volume of 1 mL. Separations were performed on a Luna C18 column (5 µm, 150 × 2.0 mm) using a linear gradient of water (A) and acetonitrile (B), each modified with 0.1% formic acid, at a flow rate of 0.4 mL/min. The gradient was programmed from 20% to 50% B over 30 min, followed by a return to initial conditions from 31 to 36 min. Absolute configurations were assigned by comparison of retention times with those of L-FDVA-

derivatized authentic amino acid standards prepared under identical conditions. Using this approach, all proteinogenic amino acid residues were unambiguously assigned. For the bishomologated tyrosine (bHtyr) residue present in compound **1**, an authentic D-bHtyr standard was not available. To resolve its configuration, two aliquots of compound **1** (0.25 mg each) were independently hydrolyzed as described above and derivatized using 1% solutions of *N*-α-(2,4-dinitro-5-fluorophenyl)-L-leucinamide (L-FDLA) and *N*-α-(2,4-dinitro-5-fluorophenyl)-D-leucinamide (D-FDLA) in acetone, respectively. The resulting derivatives were analyzed by LC-MS using the same column, with a modified gradient elution from 20% to 80% B over 30 min, followed by re-equilibration to initial conditions from 31 to 36 min. Configuration was assigned based on the interaction of the derivatives with the reversed-phase column chemistry as has been previously described in cyanopeptide analysis.[1]

Tables and Figures

Table S1. NMR Data for Micropeptin 1010 (**1**).

Table S2. NMR data for micropeptin 966 (D-Gln) (**2**).

Table S3. Molecular formulas, exact masses, and high-resolution mass spectrometric data for newly identified micropeptins and other cyanopeptides and confidence level of identification.

Table S4. Sequences of micropeptin variants isolated/identified in this study (BTA=butanoic acid, HA=hexanoic acid).

Table S5. Marfey's derivatization data and assignments for **1–3.**

Table S6. Retention times of micropeptin stereoisomers and field sample peaks under LC-MSD conditions.

Figure S1. Mirror plot of MS/MS spectra of micropeptin 950 and micropeptin 996.

Figure S2. HRMS measurement (*m/z* 973.4456) and annotated MS/MS fragmentation pattern of micropeptin 950.

Figure S3. Annotated MS/MS spectrum of micropeptin 950.

Figure S4. Annotated MS/MS spectrum of micropeptin 1005.

Figure S5. Annotated MS/MS spectrum of micropeptin 980.

Figure S6. Annotated MS/MS spectrum of micropeptin 946.

Figure S7. Annotated MS/MS spectrum of micropeptin 1038.

Figure S8. Annotated MS/MS spectrum of micropeptin 1024.

Figure S9. Isolation and characterization of [Leu^{1}]MC-LR.

Figure S10. ^{1}H NMR (500 MHz, MeOH-d_4) of [Leu^{1}]MC-LR.

Figure S11. Mass spectrometry data of **1**.

Figure S12. ^{1}H NMR (500 MHz, DMSO-d_6) of micropeptin 1010 (**1**).

Figure S13. ^{13}C NMR (125 MHz, DMSO-d_6) of micropeptin 1010 (**1**).

Figure S14. Multiplicity-edited HSQC of micropeptin 1010 (**1**).

Figure S15. HMBC of micropeptin 1010 (**1**).

Figure S16. TOCSY of micropeptin 1010 (**1**).

Figure S17. NOESY of micropeptin 1010 (**1**).

Figure S18. ^{1}H NMR (500 MHz, DMSO-d_6) of micropeptin 966 (D-Gln) (**2**).

Figure S19. Multiplicity-edited HSQC of micropeptin 966 (D-Gln) (**2**).

Figure S20. TOCSY of micropeptin 966 (D-Gln) (**2**).

Figure S21. NOESY of micropeptin 966 (D-Gln) (**2**).

Figure S22. Mass spectrometry data of **2.**

Figure S23. HRMS of ferintoic acid C *m/z* 899.4128 $[M+H]^+$.

Figure S24. ^{1}H NMR (500 MHz, DMSO-d_6) of ferintoic acid C (**3**).

Figure S25. Multiplicity-edited HSQC of ferintoic acid C (**3**).

Figure S26. TOCSY of ferintoic acid C (**3**).

Figure S27. NOESY of ferintoic acid C (**3**).

Figure S28. LC-MS analysis of the hydrolysate of **1** reacted with L-FDLA (top panel) and a mixture of L- and D-FDLA (bottom panel) to determine the configuration of the bHtyr in **1**.

Figure S29. LC-MS analysis of the hydrolysate of **2**.

Figure S30. Activity of micropeptin 996 (L-Gln), micropeptin 1010 (**1**), and micropeptin 966 (D-Gln) (**2**) against human neutrophil elastase.

Figure S31. Microcystin cluster in MS/MS molecular network subjected to two different product ion searches.

Figure S32. MS/MS cluster of anabaenopeptins/ferintoic acids annotated via product ion searching.

Figure S33. Microviridin cluster annotated using product ion searching in MS/MS networks.

Figure S34. Microginin cluster annotated using product ion searching in MS/MS networks.

Figure S35. Blast hit to the *ociB* gene (cyanopeptolin biosynthetic pathway) in the metagenomic sequence data from Lake Erie (Miller Road Park).

Table S1. NMR Data for Micropeptin 1010 (**1**) (500 MHz for ^{1}H NMR, 125 MHz for ^{13}C NMR; DMSO-d_6)

Position	δC, mult	δH, mult, J (Hz)	TOCSY	ROESY
**Val-1**				
2	55.3, CH	4.73, ovlp	3, NH	
3	30.3, CH	2.07, m	2, 4, 5, NH	
4	18.9, CH_3	0.87, ovlp	3, 5, NH	
5	16.8, CH_3	0.73, d (6.4)	3, 4, NH	
NH		7.41, ovlp	2, 3, 4, 5	*N*-MePhe-2, *N*-Me
***N*-MePhe-1**				
2	60.1, CH	5.03, ovlp	3a, 3b	Val-NH, Phe-2
3a	33.3, CH_2	3.23, m	2	
3b		2.84, m	2	
4				
5/9	129.1, CH	7.25, d (7.3)	6, 7, 8	Phe-2
6/8	128.3, CH	7.40, t (7.3)	5, 7, 9	
7	126.3, CH	7.31, t (7.2)	5, 6, 8, 9	
N-Me	30.0, CH_3	2.79, s		Val-NH
**Phe-1**	170.8, C			
2	49.7, CH	4.73, ovlp	3a, 3b	*N*-MePhe-2, 5, 9, Ahp-5
3a	34.9, CH_2	2.83, m	2, 3b	Ahp-5
3b		1.65, m	2, 3a	Ahp-5
4				
5/9	129.1, CH	6.78, d (7.4)	6, 7, 8	Ahp-5
6/8	127.5, CH	7.18, t (7.3)	5, 7, 9	
7	125.9, CH	7.13, d (7.4)	5, 6, 8, 9	
**Ahp-1**				
2	48.2, CH	3.58, m	3a, 3b, 4a, 4b, 5, NH	Ahp-2, 3b, 4b
3a	21.2, CH_2	2.38, m	2, 3b, 4a, 4b, 5, NH	Ahp-NH
3b		1.56, m	2, 3a, 4b, NH	Ahp-2
4a	28.7, CH_2	1.66, m	2, 3a, 4b, 5, NH	Ahp-3a, 5
4b		1.48, m	2, 3a, 4a, 5, NH	Ahp-2, 5
5	73.2, CH	5.03, ovlp	2, 3a, 4a, 4b, OH	Ahp-4a, 4b, Phe-2, 3a, 5, 9
NH		7.10, ovlp	2, 3a, 3b, 4a, 4b	Ahp-3a, bHtyr-2, bHtyr-NH
OH		6.05, br	5	
**bHtyr-1**				
2	51.3, CH	4.22, m	3, 4, 5a, 5b, NH	Ahp-NH
3	29.1 CH_2	1.83, m	2, 4, 5a, 5b, NH	
4	27.1 CH_2	1.41, m	2, 3, 5a, 5b, NH	
5a	33.4, CH_2	2.40, m	2, 3, 4, 5b	
5b		2.35, m	2, 3, 4, 5a	
6	155.8, C			
7/9	128.9, CH	6.91, d (8.2)	7, 9	
8/10	114.7, CH	6.64, d (8.2)	6, 8	
11				
NH		8.42, d (8.7)	2, 3, 4	Ahp-NH, Thr-2, 3
**Thr-1**				
2	54.2, CH	4.62, m	NH	bHtyr-NH
3	71.6, CH	5.41, m	4	bHtyr-NH
4	17.3, CH_3	1.17, d (6.4)	3	
NH		7.91, d (9.2)	2	Gln-2, 3a
**Gln-1**				
2	51.7, CH	4.37, m	3a, 3b, 4, NH	Thr-NH
3a	27.3, CH_2	1.90, m	2, 3b, 4, NH	Thr-NH
3b		1.71, m	2, 3a, 4, NH	
4	31.4, CH_2	2.17, m	2, 3a, 3b, NH	

5				
NH		8.05, d (7.7)	2, 3a, 3b, 4	BTA-2
NH_2		7.27, br<br>6.75, br		
**BTA-1**				
2	36.6, CH_2	2.12, m	3, 4	Gln-NH
3	18.4. CH_2	1.53, m	2, 4	
4	13.3, CH_3	0.88, ovlp	2, 3	

Table S2. NMR data for micropeptin 966 (D-Gln) (**2**) (500 MHz for ^{1}H NMR, 125 MHz for ^{13}C NMR; DMSO-d_6).

Position	δC, mult	δH, mult, *J* (Hz)	TOCSY	NOESY
**Val-1**				
2	54.2, CH	4.74, m	3, 4, 5, NH	
3	30.3, CH	2.07, m	2, 4, 5, NH	
4	18.7, CH_3	0.86, d (7.3)	2, 3, 5, NH	
5	16.8, CH_3	0.72, d (6.8)	2, 3, 4, NH	
NH		7.44, ovlp[a]	2, 3, 4, 5	*N*-MePhe-2
***N*-MePhe-1**				
2	60.6, CH	5.04, m	3a, 3b	Val-NH, Phe-2, 3a
3a	33.6, CH_2	3.23, m	2	
3b		2.87, m	2	
4				
5/9	129.2, CH	7.25, d (7.5)	6, 7, 8	Phe-3a
6/8	128.3, CH	7.42, t (7.5)	5, 7, 9	
7	125.7, CH	7.32, d (7.4)	5, 6, 8, 9	
N-Me	30.0, CH_3	2.79, s		
**Phe^1-1**				
2	49.5, CH	4.75, ovlp	3a, 3b	*N*-MePhe-2, Ahp-5, NH
3a	34.7, CH_2	2.83, m	2	*N*-MePhe-5, 9, Ahp-5
3b		1.67, m	2	Ahp-5
4				
5/9	129.2, CH	6.78, d (7.6)	6, 7, 8	
6/8	127.5, CH	7.17, t (7.3)	5, 7, 9	
7	125.9, CH	7.13, d (7.3)	5, 6, 8, 9	
**Ahp-1**				
2	48.4, CH	3.60, m	3a, 3b, 4a, NH	Ahp-3a, 3b, Ahp-NH
3a	20.9, CH_2	2.40, m	2, 3b, 4a, 4b, 5, NH	Ahp-NH
3b		1.59, m	2, 3a	Ahp-2
4a	29.2, CH_2	1.66, ovlp	2, 3a, 4b, NH	Ahp-3a, 5, OH
4b		1.51, m	4a, 5, NH	Ahp-5
5	73.5, CH	5.04, ovlp	3a, 4a, 4b, OH	Ahp-4a, 4b, Phe^1-2, 3a, 3b, 4a
NH		7.10, d (9.3)	2, 3a, 4a, 4b	Ahp-2, 3a, Phe^2-NH
OH		6.06, br	5	Ahp-4a
**Phe^2-1**				
2	48.4, CH	4.32, m	3a, 3b, NH	
3a	36.9, CH_2	2.64, m	2, 3b, NH	
3b		1.79, m	2, 3a, NH	
4				
5/9	123.8, CH	7.11, ovlp	6, 8	
6/8	122.6, CH	7.08, ovlp	5, 7	
7	122.2, CH	6.91, m		
NH		8.59, d (8.3)	2, 3a, 3b	Ahp-NH, Thr-2, 3
**Thr-1**				
2	54.6, CH	4.53, m	NH	Phe-NH
3	71.3, CH	5.39, m	4	Phe-NH
4	17.5, CH_3	1.18, d (6.5)	3	
NH		7.91, m	2	Gln-2
**Gln-1**				
2	52.1, CH	4.41, m	3a, 3b, 4, NH	Thr-NH
3a	27.6, CH_2	1.89, m	2, 3b, 4, NH	
3b		1.75, m	2, 3a, 4, NH	
4	31.3, CH_2	2.11, m	2, 3a, 3b, NH	
5				
NH		8.10, d (8.0)	2, 3a, 3b, 4	BTA-2

NH_2		7.28, br		
		6.78, br		
**BTA-1**				
2	36.8, CH_2	2.13, m	3, 4	Gln-NH
3	18.4. CH_2	1.54, m	2, 4	
4	13.1, CH_3	0.89, d (7.3)	2, 3	

[a]overlapping signals

Table S3. Molecular formulas, exact masses, and high-resolution mass spectrometric data for newly identified micropeptins and other cyanopeptides and confidence level of identification.

Name	Molecular formula	Calcd $[M+H–H_2O]^+$	Exact mass	calcd $[M+H]^+$ or $[M+Na]^+$	found	Mass error (ppm)	Confidence Level
MP 1010	$C_{53}H_{70}N_8O_{12}$	993.5080	1010.5113	1033.5005	1033.5009	0.39	1
MP 966 (D-Gln)	$C_{51}H_{66}N_8O_{11}$	949.4818	966.4851	989.4743	989.4771	2.83	1
MP 980	$C_{52}H_{68}N_8O_{11}$	963.4975	980.5008	1003.4900	1003.4903	0.30	2
MP 950	$C_{47}H_{66}N_8O_{11}S$	933.4539	950.4572	973.4464	973.4456	-0.82	2
MP 1005	$C_{53}H_{67}N_9O_{11}$	988.4927	1005.496	1028.4852	1028.4856	0.39	2
MP 946	$C_{49}H_{70}N_8O_{11}$	929.5131	946.5164	969.5056	969.5060	0.41	2
MP 1038	$C_{55}H_{74}N_8O_{12}$	1021.5393	1038.5426	1061.5318	1061.5318	0.00	2
MP 1024	$C_{54}H_{72}N_8O_{12}$	1007.5237	1024.5270	1047.5162	1047.5167	0.48	2
[Leu^1[MC-LR]	$C_{52}H_{80}N_{10}O_{12}$		1036.5957	1037.6030	1037.6030	0.00	1
[Leu 1 ,Glu(OCH 3) 6] MC-LR	$C_{53}H_{82}N_{10}O_{12}$		1050.6114	1051.6192	1051.6188	-0.38	2
Ferintoic acid C	$C_{46}H_{58}N_8O_9S$		898.4047	899.4120	899.4128	0.89	1

Table S4. Sequences of micropeptin variants isolated/identified in this study (BTA=butanoic acid, HA=hexanoic acid).

	Residue 1	Residue 2	Residue 3	Residue 4	Residue 5	Residue 6	Residue 7	Side chain
MP 996	Val	*N*Me-Phe	Phe	Ahp	Htyr	Thr	Gln	BTA
MP 982	Val	*N*Me-Phe	Phe	Ahp	Tyr	Thr	Gln	BTA
MP 982 (L-Ser)	Val	*N*Me-Phe	Phe	Ahp	Htyr	**Ser**	Gln	BTA
MP 957	Val	***N*Me-Trp**	Phe	Ahp	**Val**	Thr	Gln	BTA
MP 1010	Val	*N*Me-Phe	Phe	Ahp	**bHtyr**	Thr	Gln	BTA
MP 966 (D-Gln)	Val	*N*Me-Phe	Phe	Ahp	**Phe**	Thr	Gln	BTA
MP 980	Val	*N*Me-Phe	Phe	Ahp	**Hphe**	Thr	Gln	BTA
MP 950	Val	*N*Me-Phe	Phe	Ahp	**Met**	Thr	Gln	BTA
MP 1005	Val	*N*Me-Phe	Phe	Ahp	**Trp**	Thr	Gln	BTA
MP 946	Val	*N*Me-Phe	Phe	Ahp	**Hleu/ HIle**	Thr	Gln	BTA
MP 1038	Val	*N*Me-Phe	Phe	Ahp	**bHtyr**	Thr	Gln	**HA**
MP 1024	Val	*N*Me-Phe	Phe	Ahp	**Htyr**	Thr	Gln	**HA**

Table S5. Marfey's derivatization data and assignments for **1–3.**

**Amino Acid**	**t_R (min)**	**Ferintoic acid C (3)**	**Micropeptin 966 (D-Gln) (2)**	**Micropeptin 1010 (1)**
L-Glutamine	13.36			
D-Glutamine	16.99			
L-Glutamic acid	15.10			15.12 (L)
D-Glutamic acid	16.61		16.64 (D) (from D-Gln)	
L-Serine	13.09			
D-Serine	13.92			
L-Tryptophan	25.11	25.14 (L)		
D-Tryptophan	28.68			
L-Valine	20.86		20.59 (L)	20.67 (L)
D-Valine	27.66			
L-*N*-Me-Phenylalanine	26.12		25.74 (L)	25.81 (L)
D-*N*-Me Phenylalanine	27.48			
L-Phenylalanine	25.44	25.09 (L)	25.00 (L)	25.12 (L)
D-Phenylalanine	30.95			
L-Homotyrosine	21.45	20.87 (L)		
D-Homotyrosine	24.37			
L-Methionine	20.52	20.96 (L)		
D-Methionine	26.22			
L-Threonine	12.86		12.47 (L)	12.54 (L)
L-allo-Threonine	13.66			
D-allo-Threonine	15.73			
D-Threonine	17.66			
L-*N*-Me-Alanine	18.57	18.66 (L)		
D-*N*-Me-Alanine	18.98			
L-Lysine	6.20			
D-Lysine	5.56	5.58 (D)		
Bis-homotyrosine (L-FDLA)	17.61			(L)
Bis-homotyrosine (D-FDLA)	18.89			

Table S6. Detection of micropeptins in Lake Erie samples. Retention times of micropeptin stereoisomers and field sample peaks under LC-MSD conditions (Luna C18, 150 × 2 mm, 5 µm, 55% A / 45% B, 0.6 mL/min).

**Sample / Standard**	**Name**	***m/z***	**t_R (min)**
Standard	Micropeptin 982 (L-Gln)	1005	1.80
Standard	Micropeptin 982 (L-Ser)	1005	2.56
Standard	Micropeptin 982 (D-Gln)	1005	2.72
Standard	Micropeptin 982 (L-*allo*-Thr)	1005	2.90
Field Extract (Huntington)	Micropeptin 982 (L-Gln)	1005	1.86
Standard	Micropeptin 996 (L-Gln)	1019	2.75
Standard	Micropeptin 996 (D-Gln)	1019	2.86
Field Extract (Huntington)	Micropeptin 996 (L-Gln)	1019	2.78

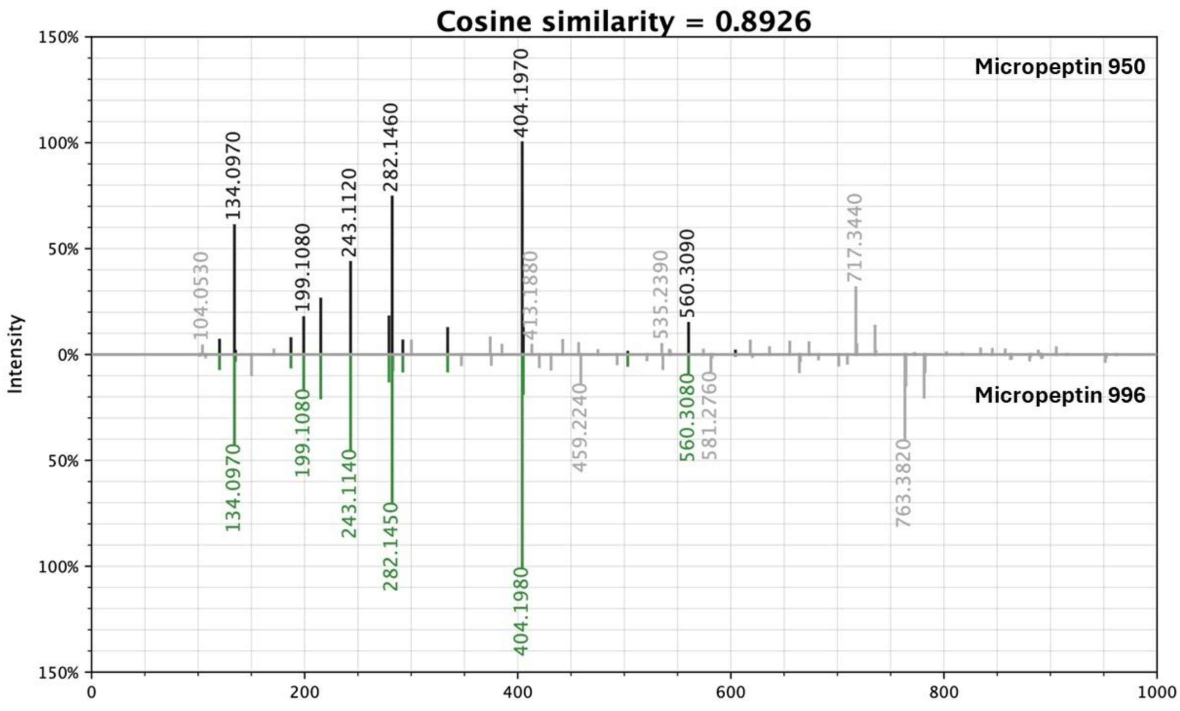

Figure S1. Mirror plot of MS/MS spectra of micropeptin 950 and micropeptin 996.

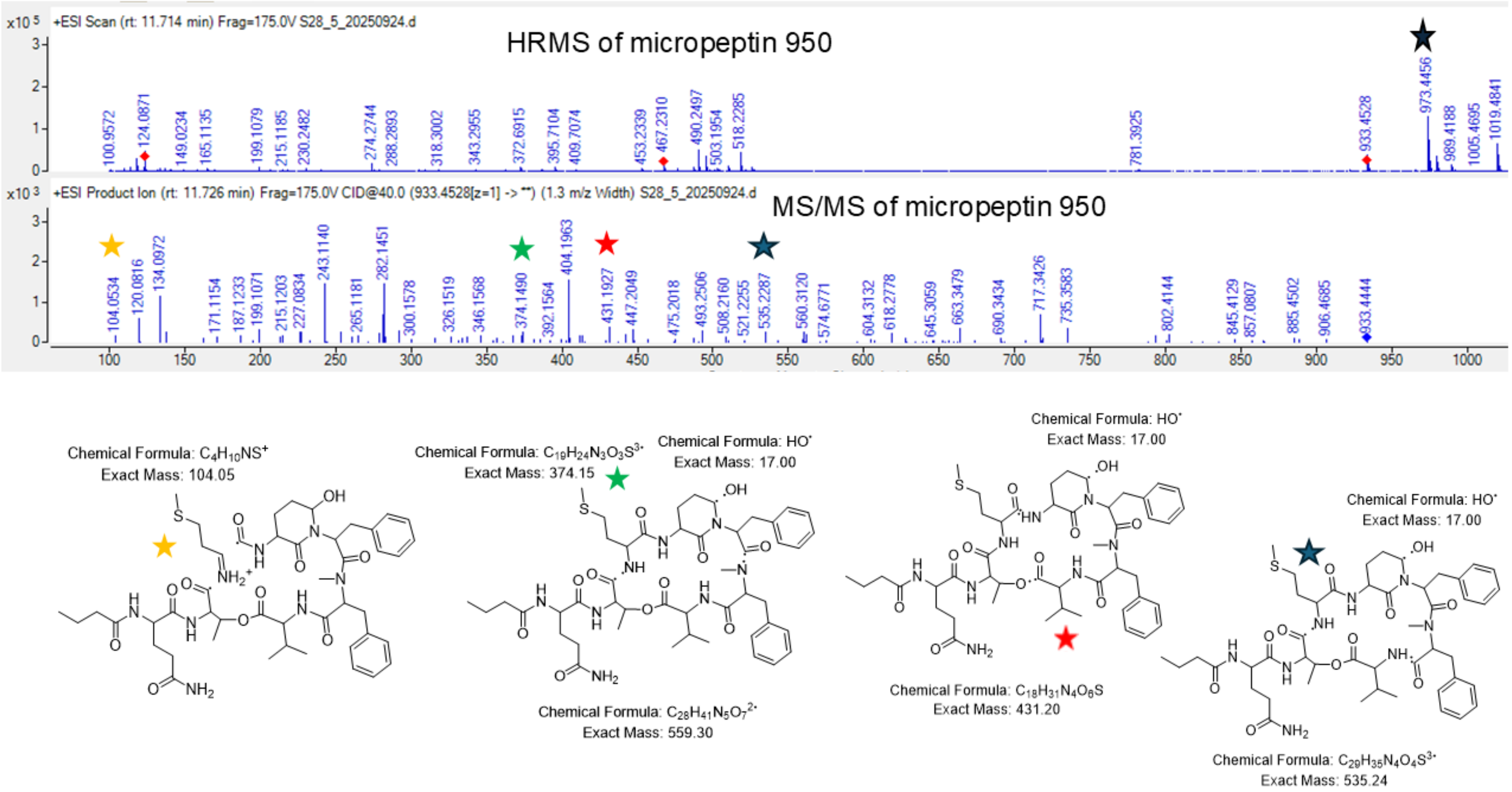

Figure S2. HRMS measurement (*m/z* 973.4456) and annotated MS/MS fragmentation pattern of micropeptin 950.

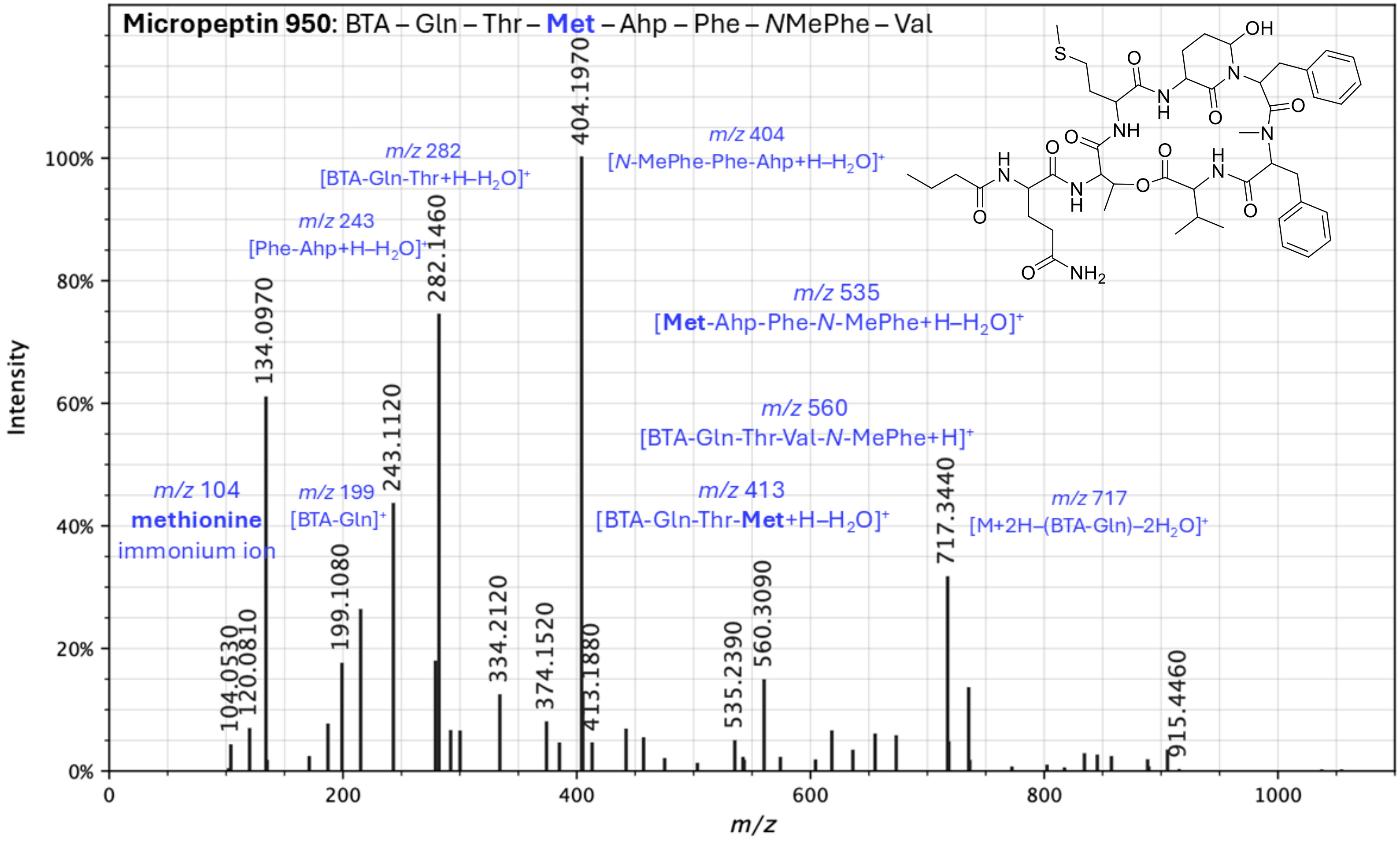

Figure S3. Annotated MS/MS spectrum of micropeptin 950.

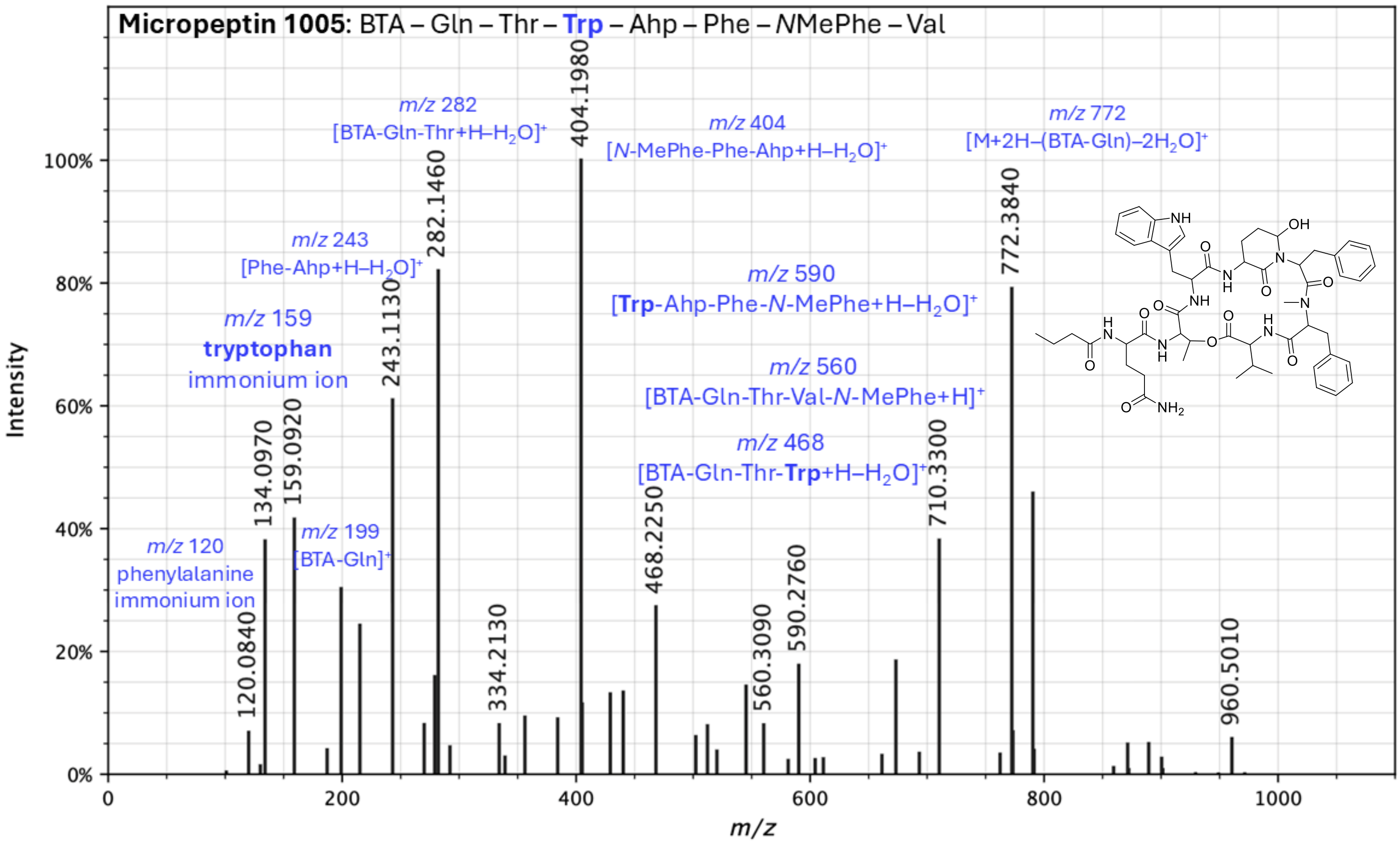

Figure S4. Annotated MS/MS spectrum of micropeptin 1005.

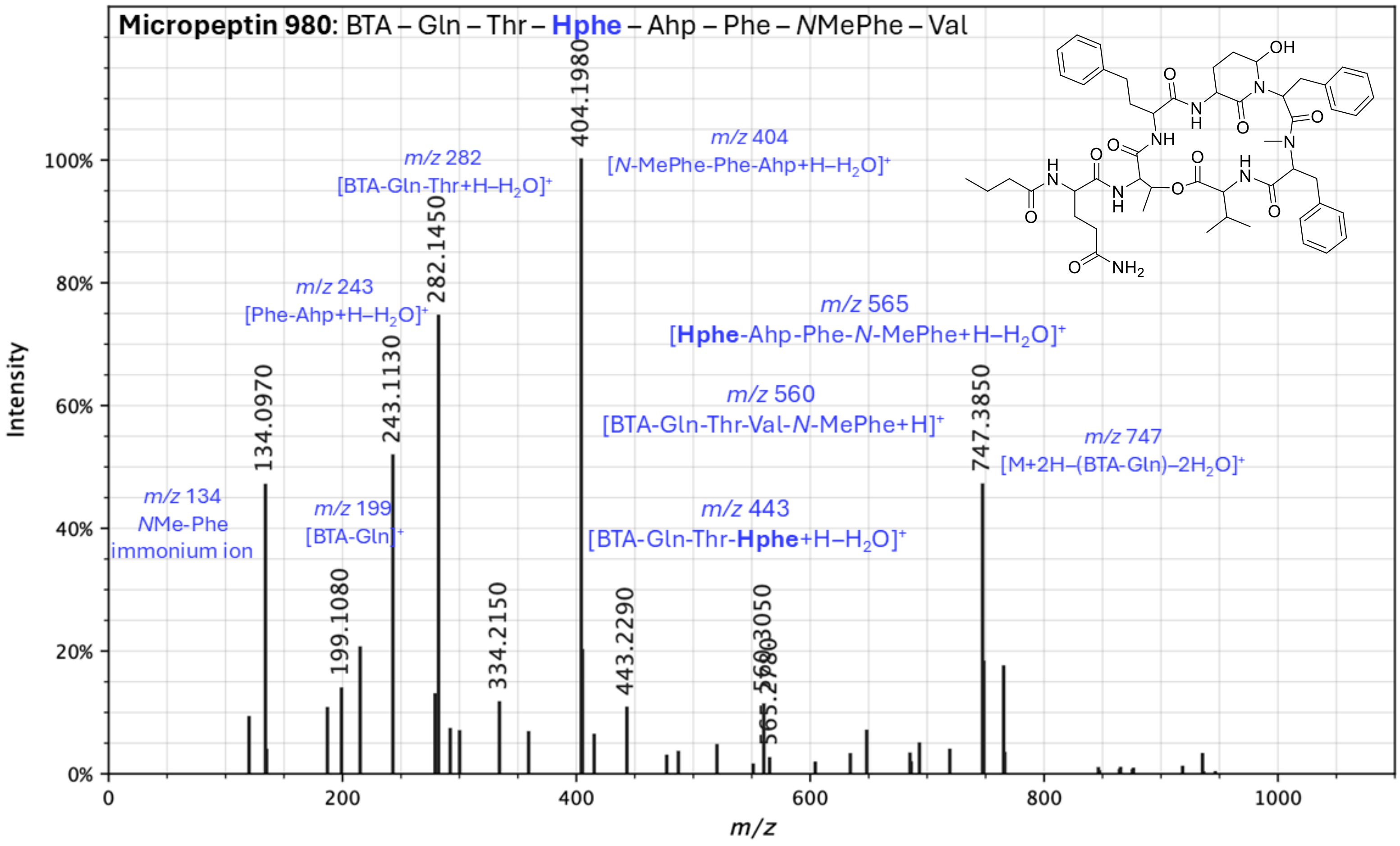

Figure S5. Annotated MS/MS spectrum of micropeptin 980.

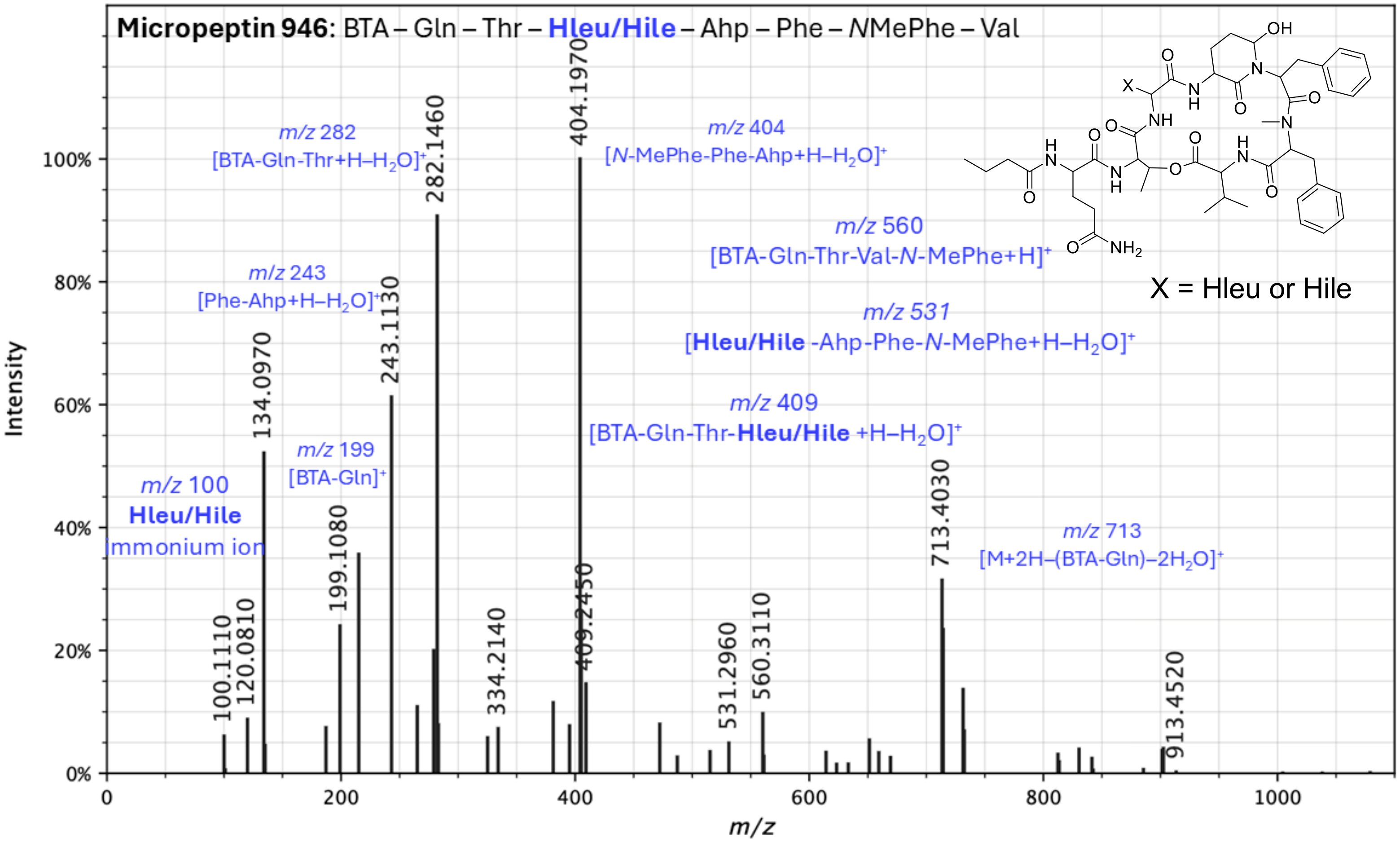

Figure S6. Annotated MS/MS spectrum of micropeptin 946.

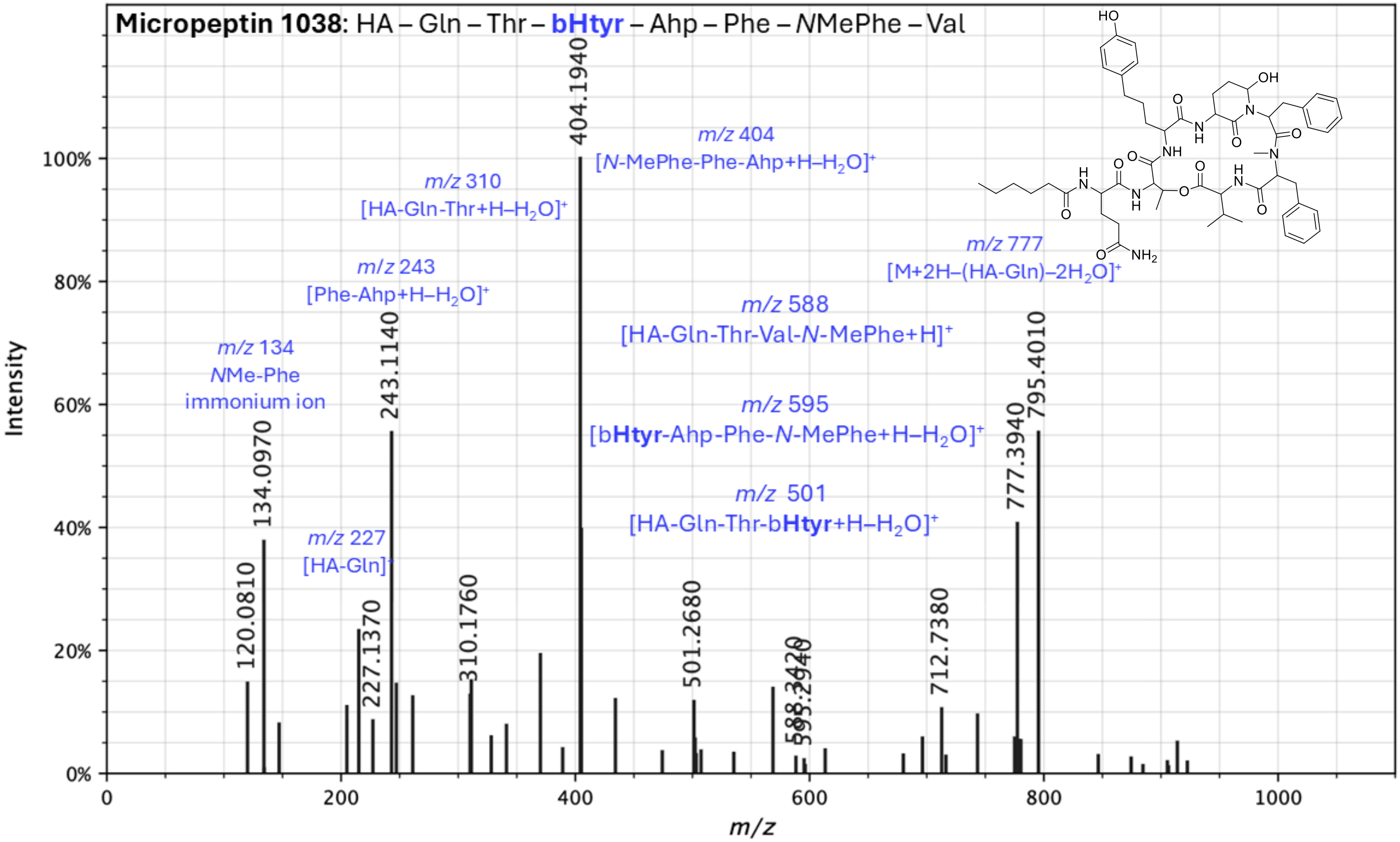

Figure S7. Annotated MS/MS spectrum of micropeptin 1038.

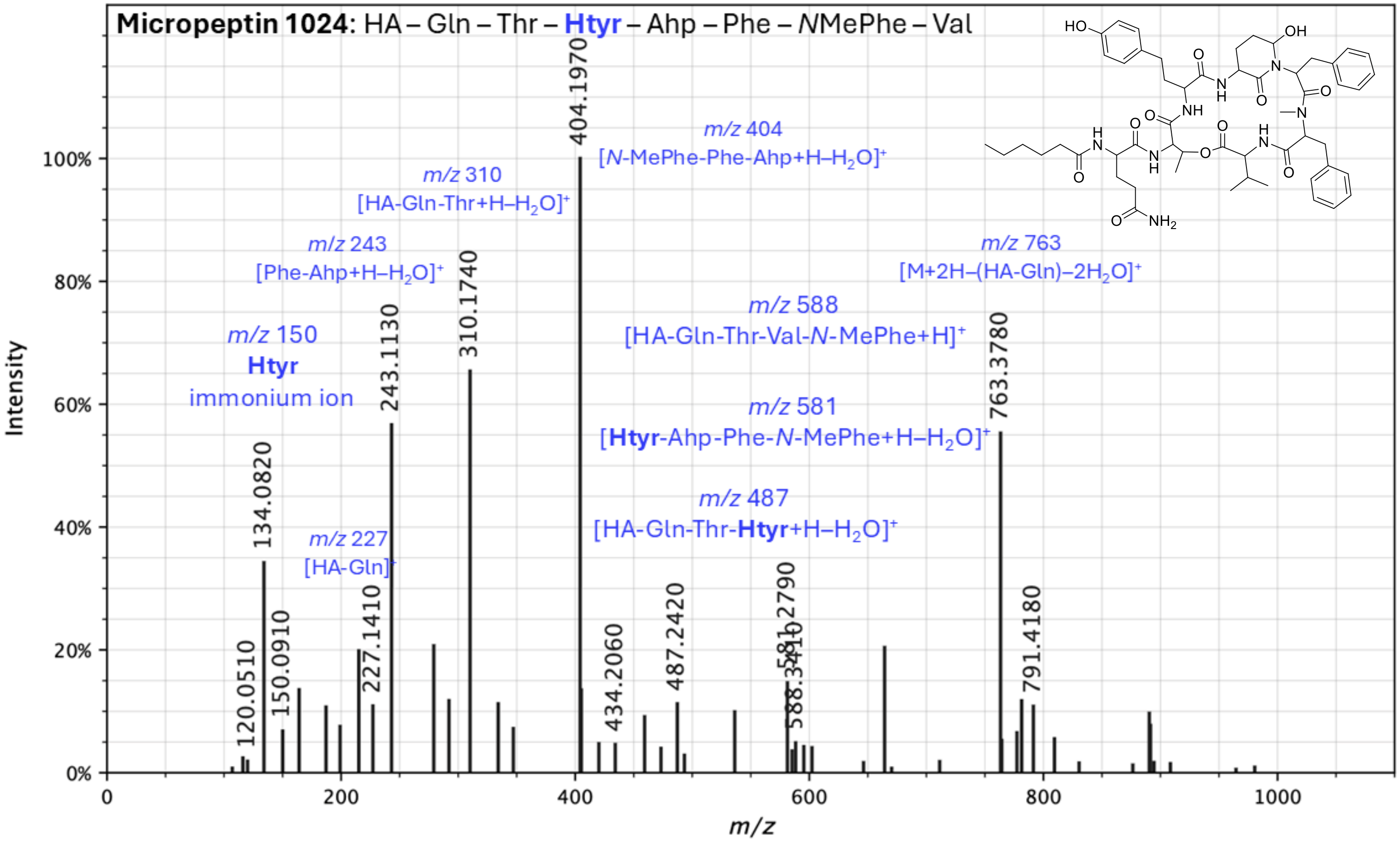

Figure S8. Annotated MS/MS spectrum of micropeptin 1024.

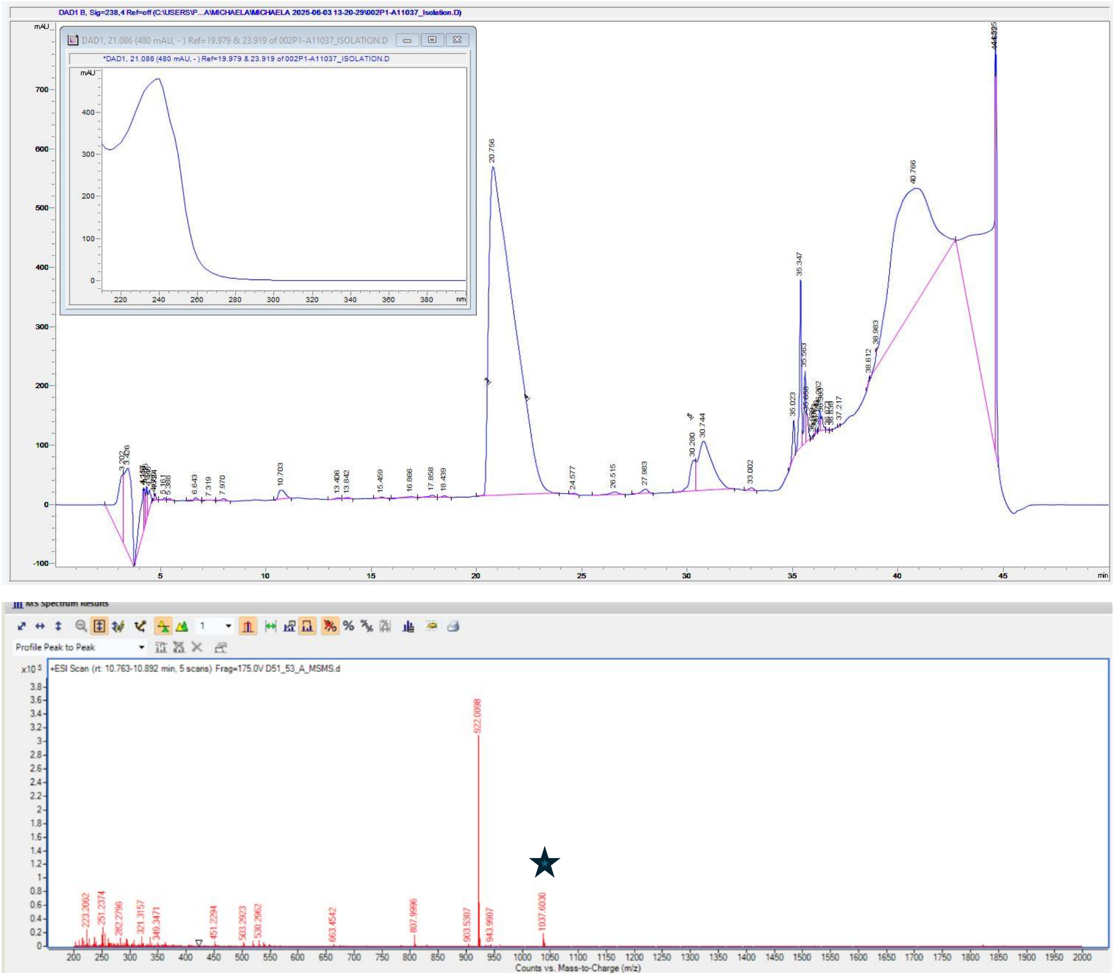

Figure S9. Isolation and characterization of [Leu^1]MC-LR. Top panel: HPLC-DAD analysis with UV spectrum with λ_{max} of 238 nm (inset) of [Leu^1]MC-LR. Bottom panel: LC-HRMS analysis of [Leu^1]MC-LR with *m/z* 1037.6030 (star).

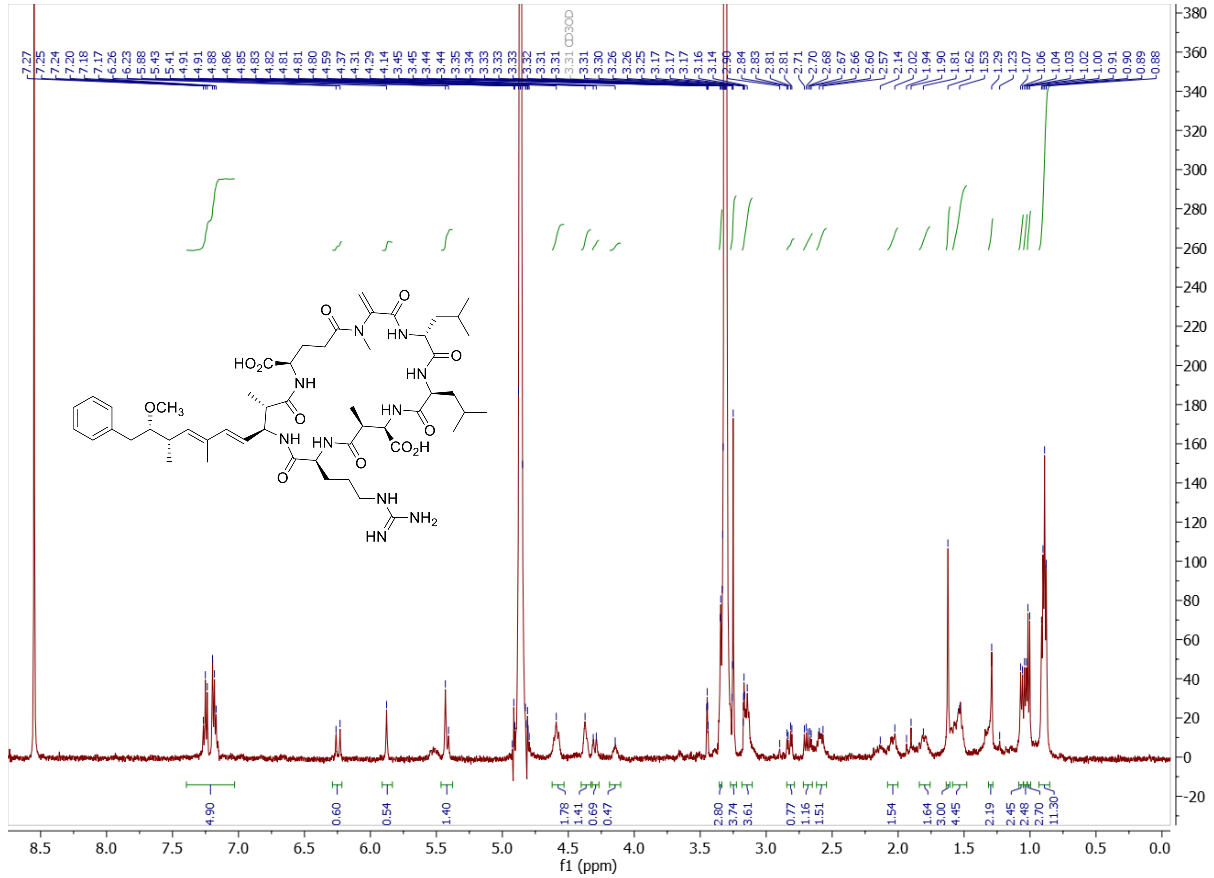

Figure S10. ^{1}H NMR (500 MHz, MeOH-d_4) of [Leu[1]]MC-LR.

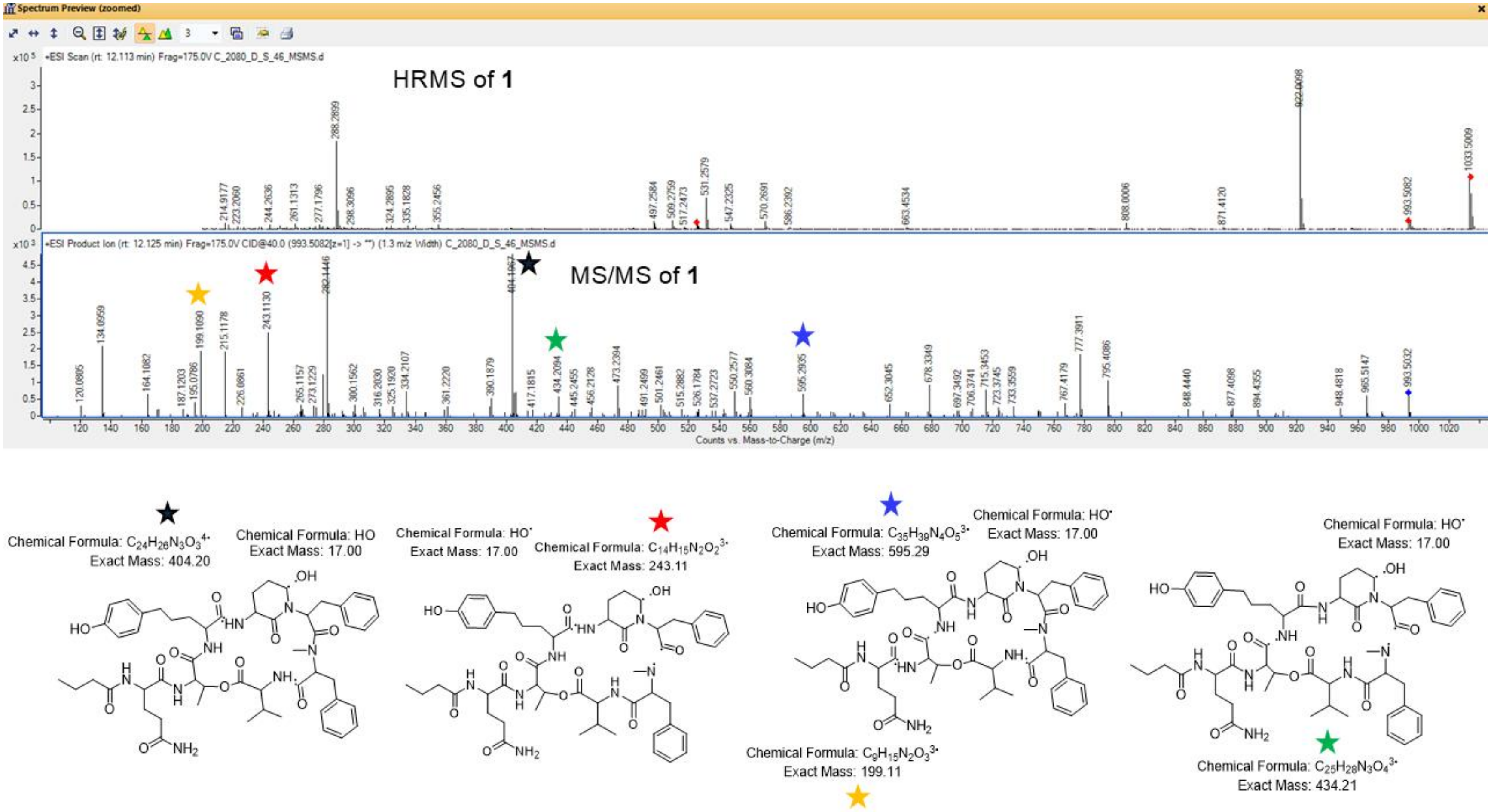

Figure S11. Mass spectrometry data of **1**. Top panel: HRMS of compound **1** *m/z* 1033.5009 $[M+Na]^+$. Middle panel: MS/MS of **1** with key fragmentation ions noted with stars, which correspond to the putative fragmentations illustrated in the bottom panel.

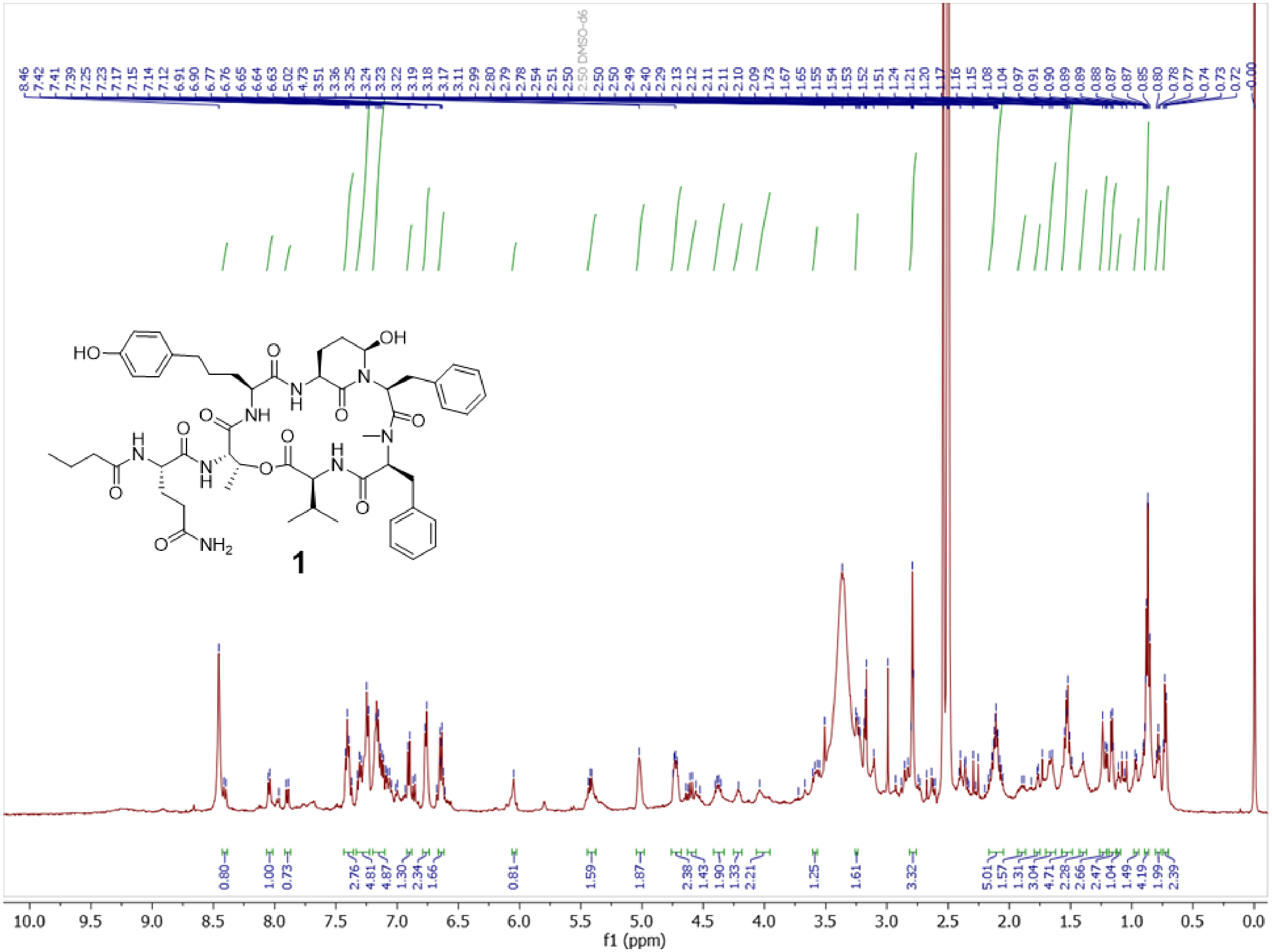

Figure S12. ^{1}H NMR (500 MHz, DMSO-d_6) of micropeptin 1010 (**1**).

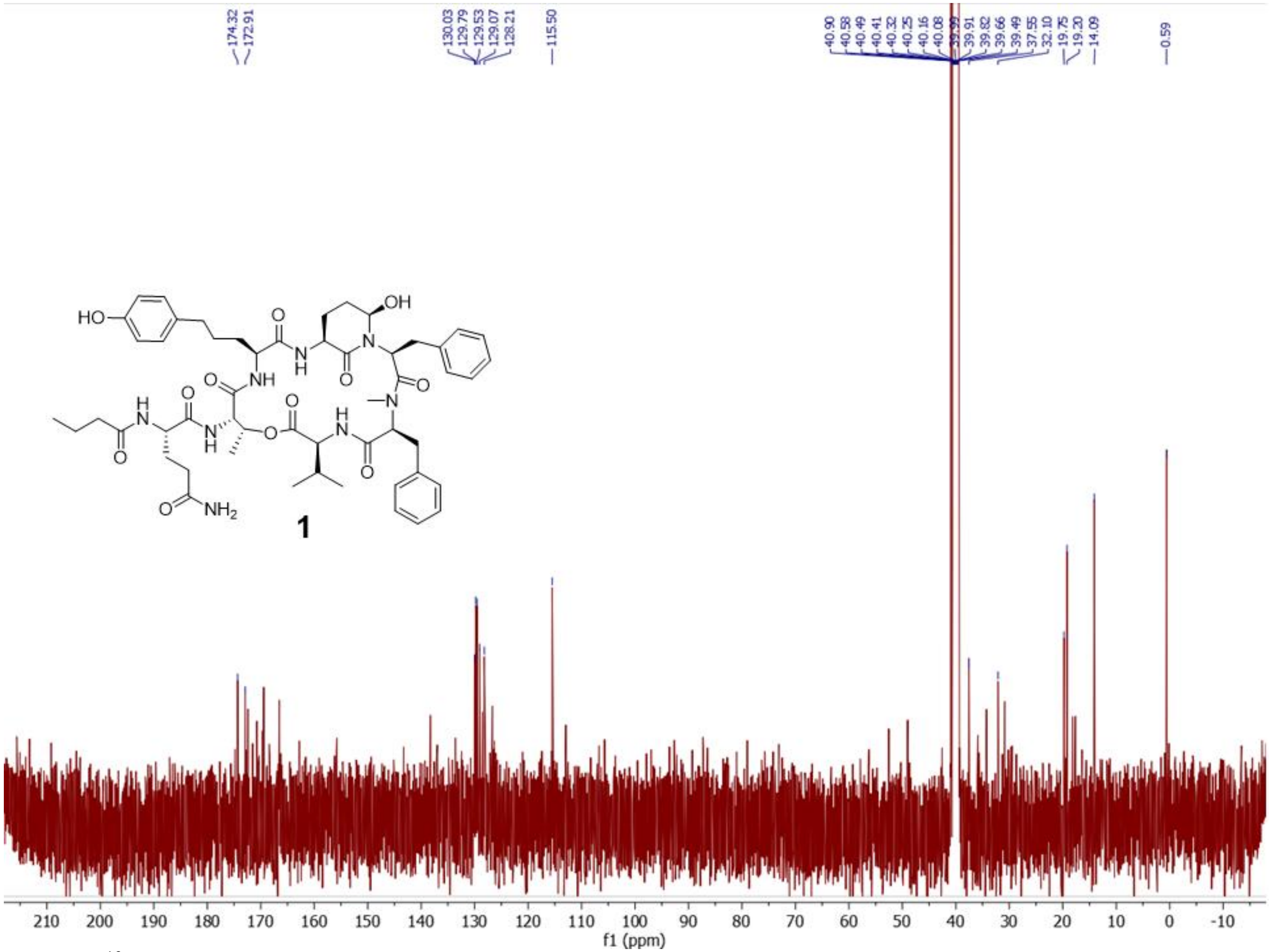

Figure S13. ^{13}C NMR (125 MHz, DMSO-d_6) of micropeptin 1010 (**1**).

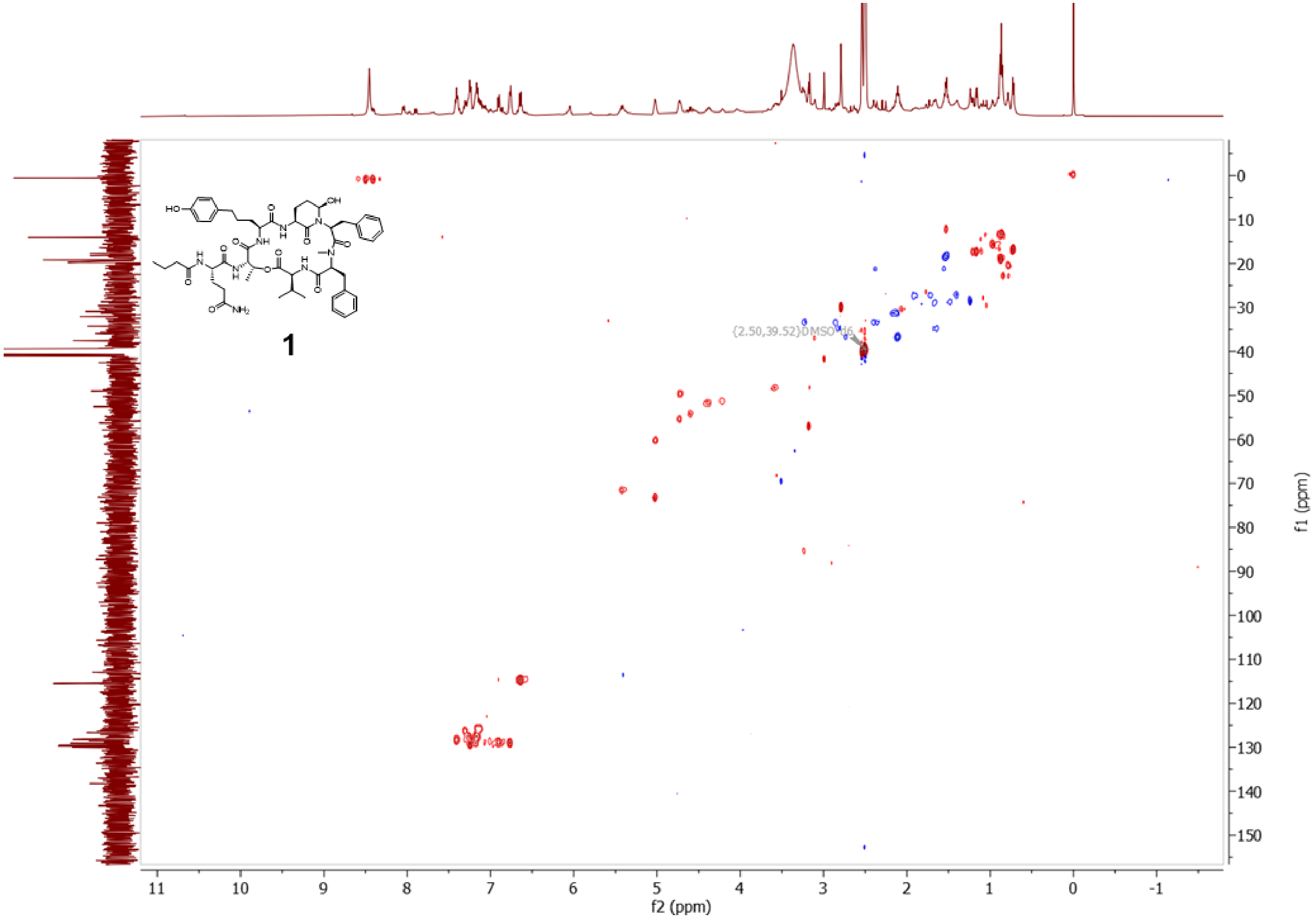

Figure S14. Multiplicity-edited HSQC of micropeptin 1010 (**1**).

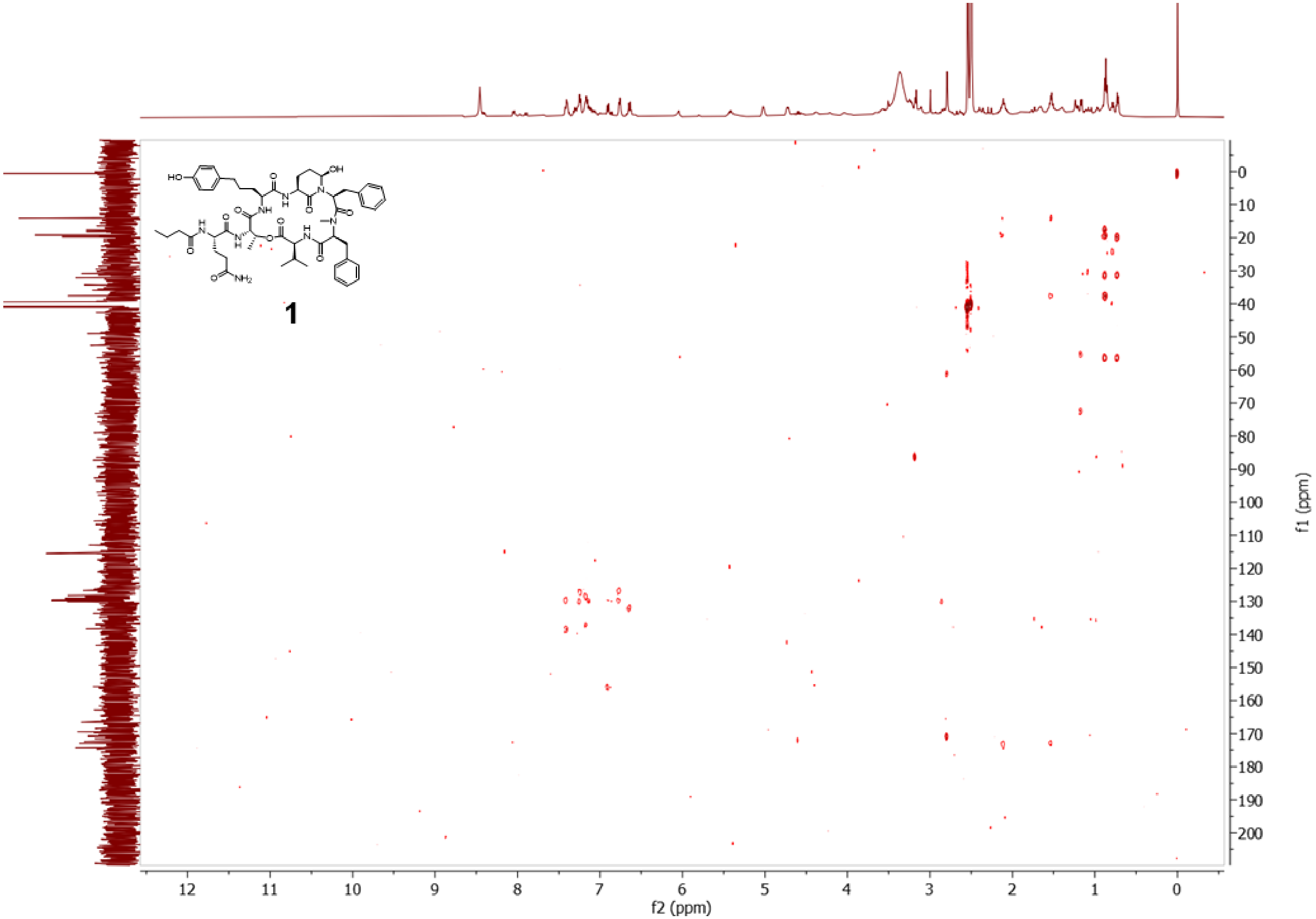

Figure S15. HMBC of micropeptin 1010 (**1**).

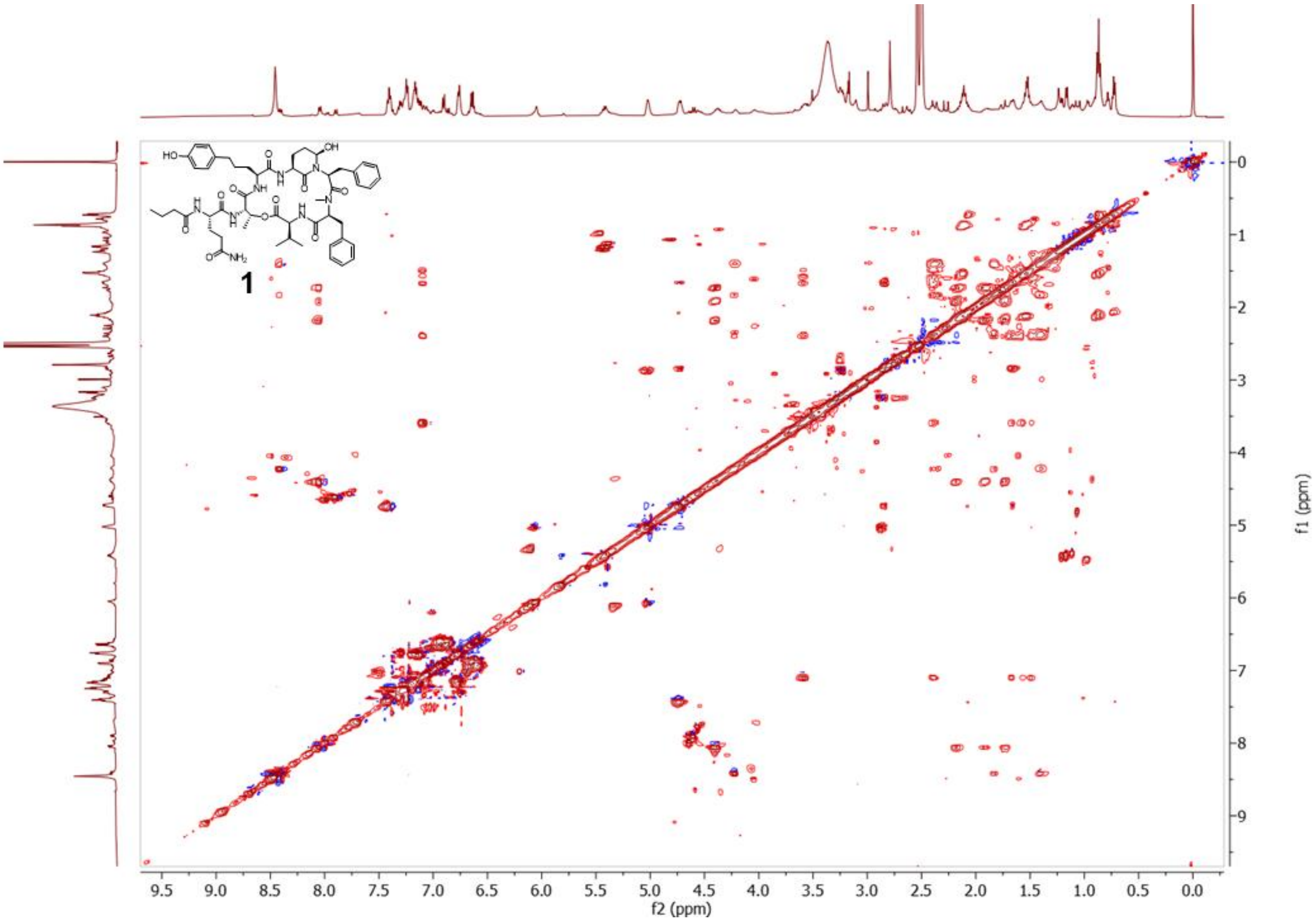

Figure S16. TOCSY of micropeptin 1010 (**1**).

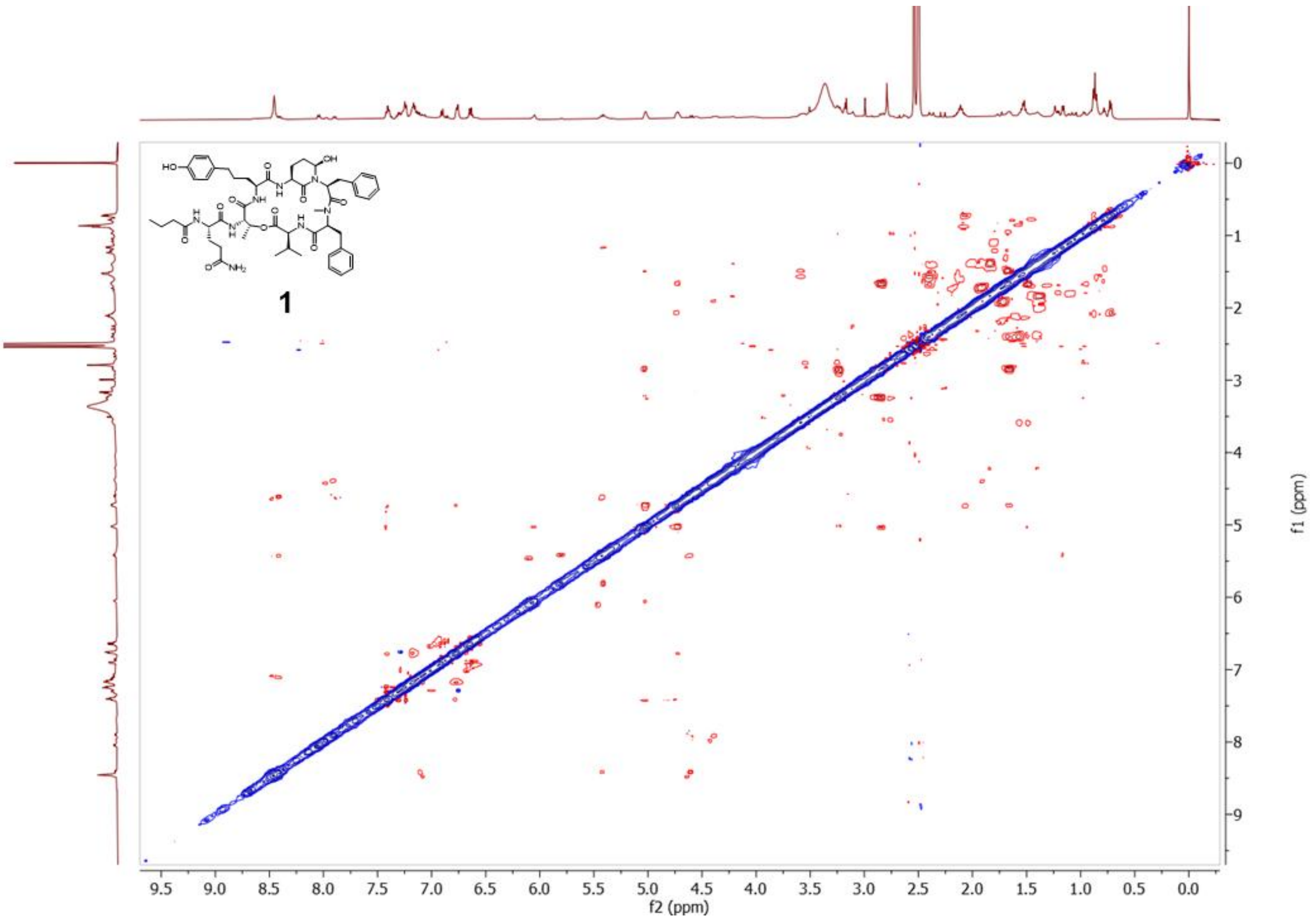

Figure S17. NOESY of micropeptin 1010 (**1**).

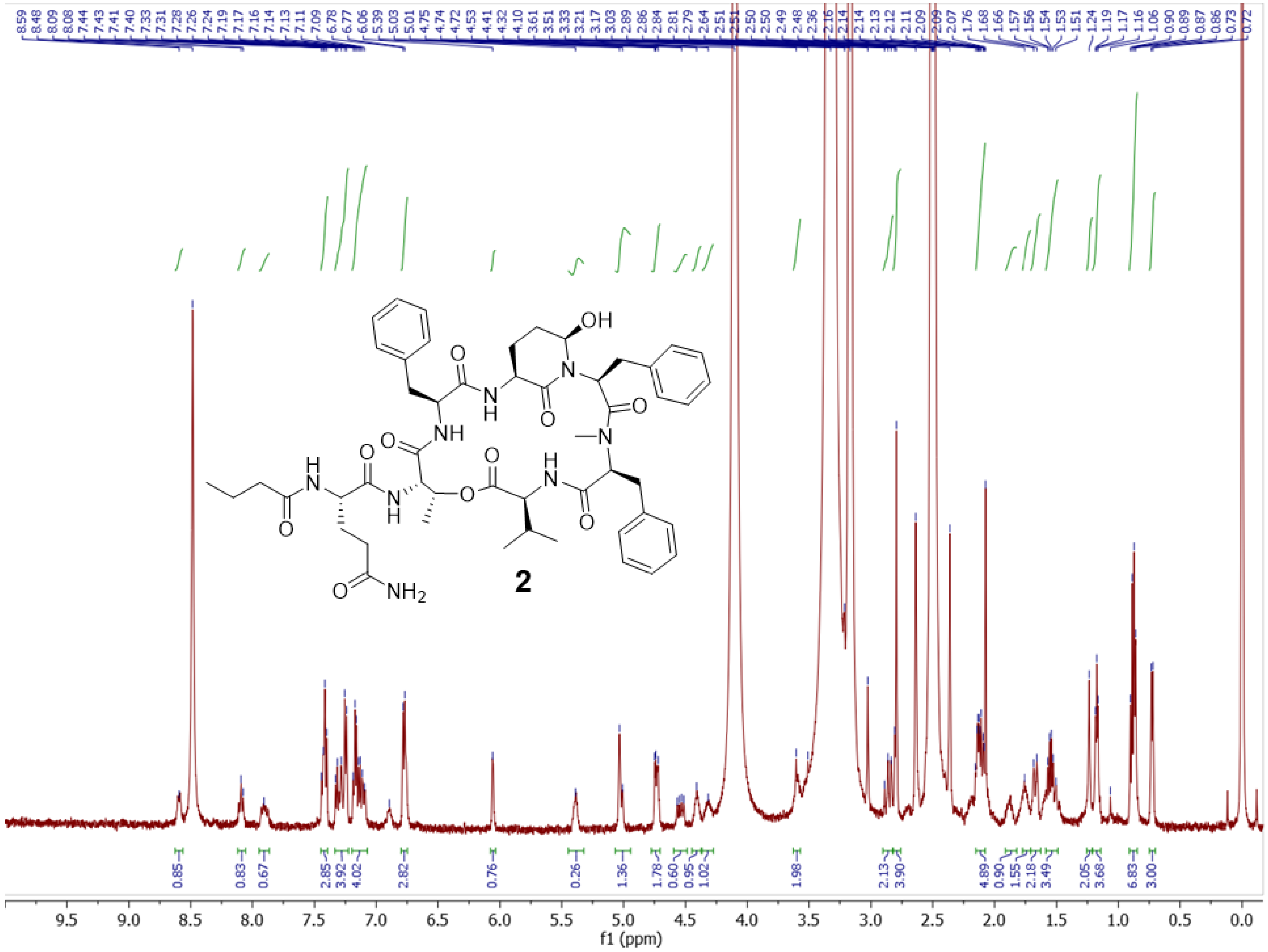

Figure S18. ^{1}H NMR (500 MHz, DMSO-d_6) of micropeptin 966 (D-Gln) (**2**).

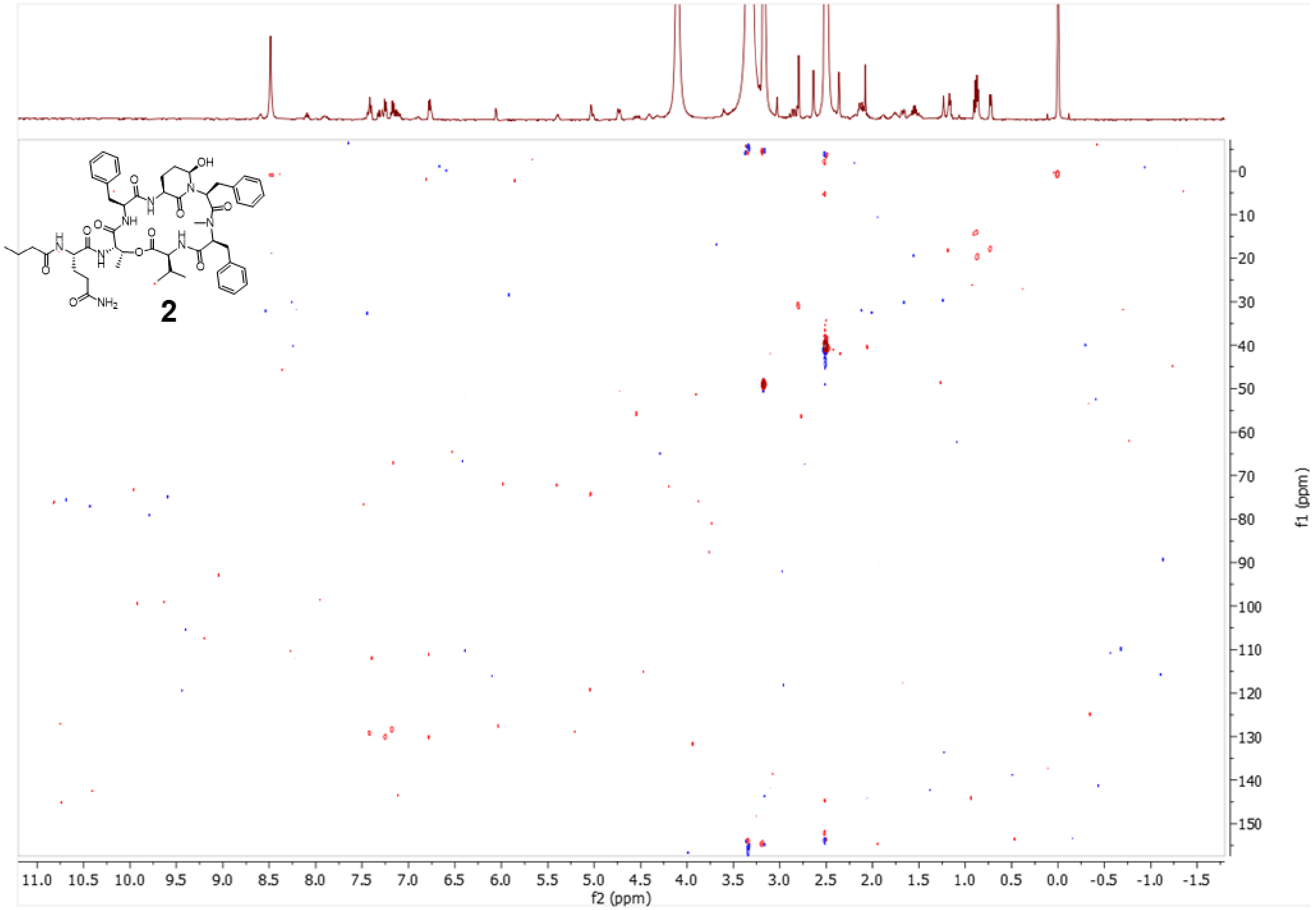

Figure S19. Multiplicity-edited HSQC of micropeptin 966 (D-Gln) (**2**).

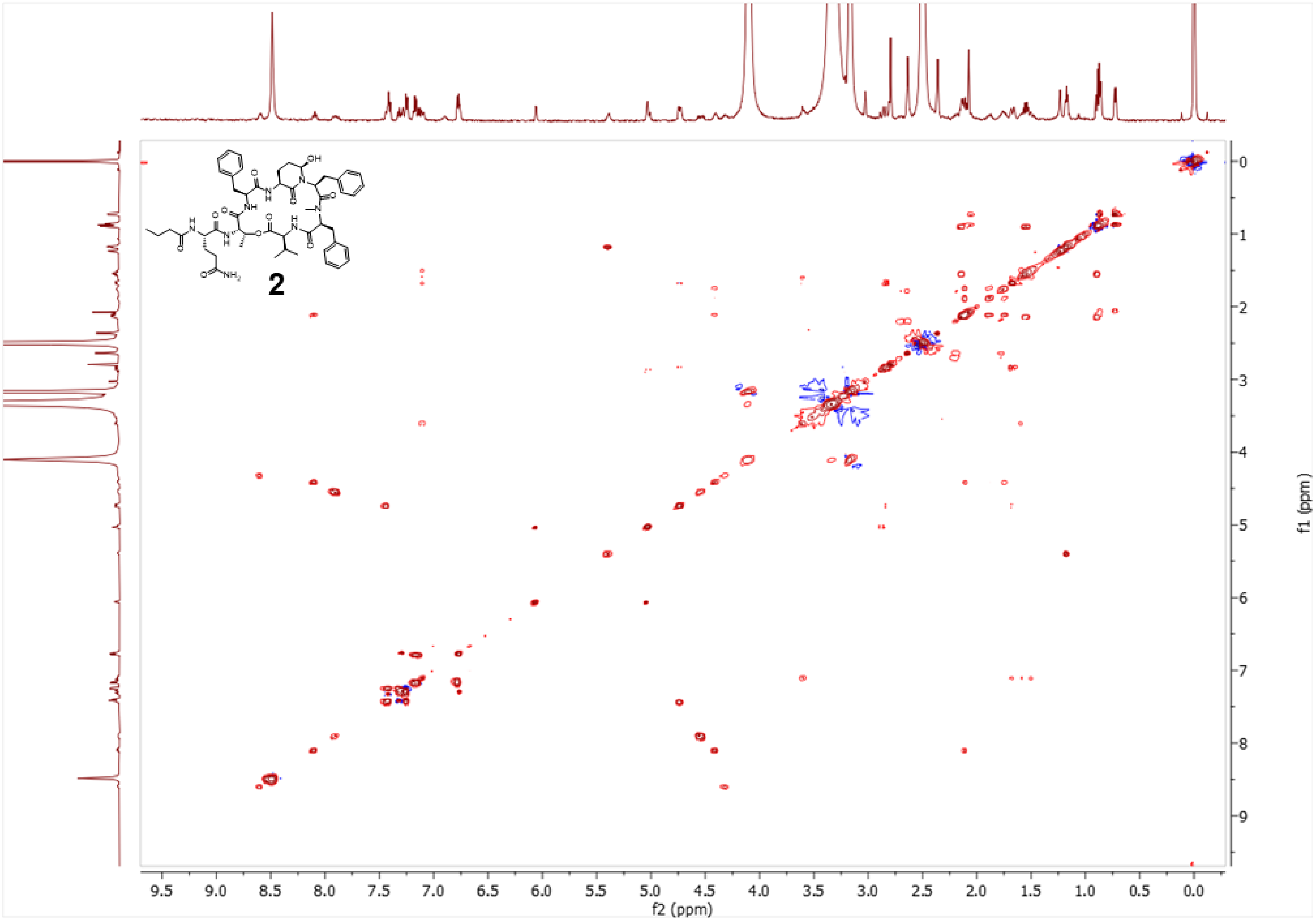

Figure S20. TOCSY of micropeptin 966 (D-Gln) (**2**).

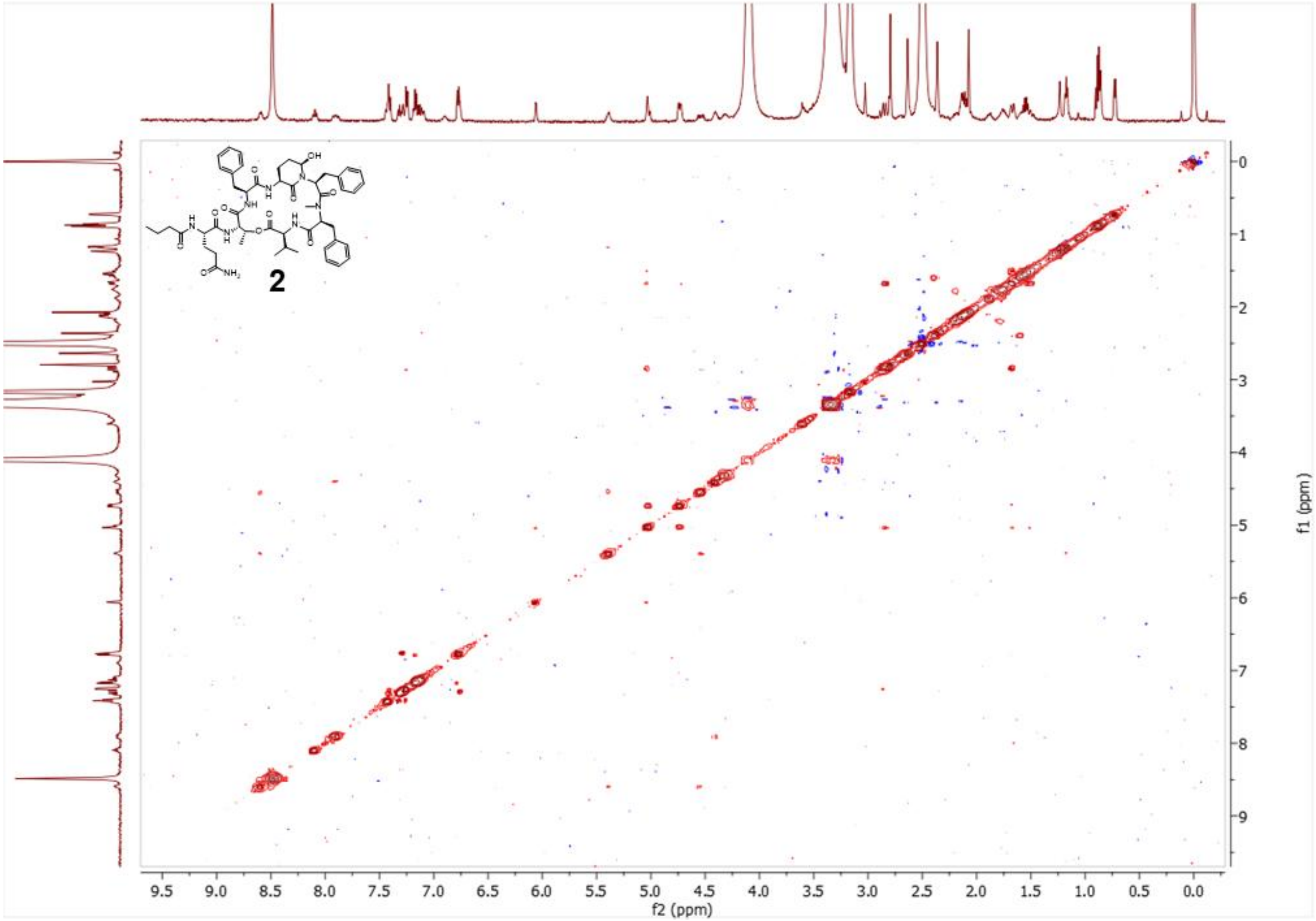

Figure S21. NOESY of micropeptin 966 (D-Gln) (**2**).

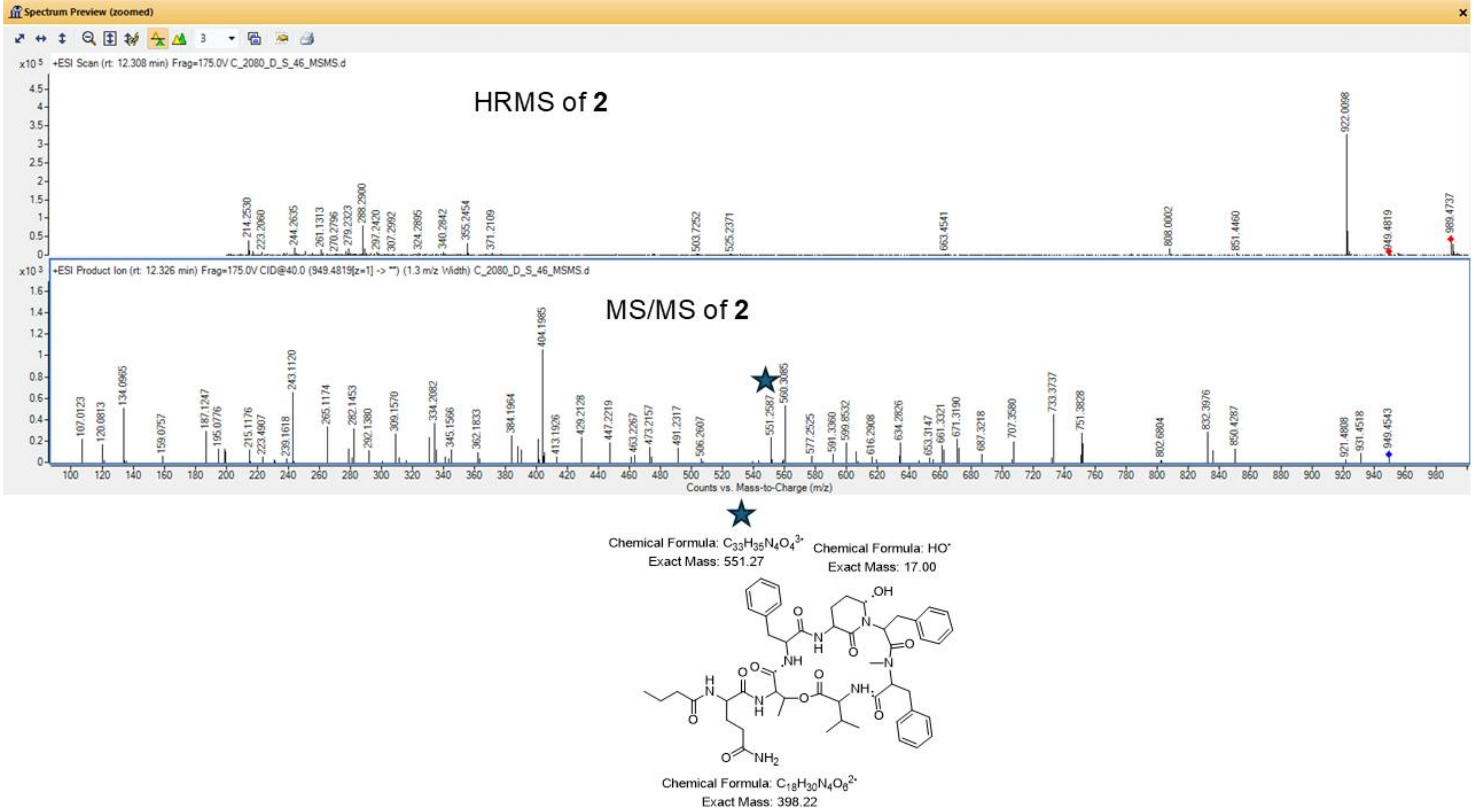

Figure S22. Mass spectrometry data of **2.** Top panel: HRMS of compound **2** *m/z* 989.4737 [M+Na]$^+$. Middle panel: MS/MS of **2** with a key fragmentation ion noted with a star, which corresponds to the putative fragmentation illustrated in the bottom panel.

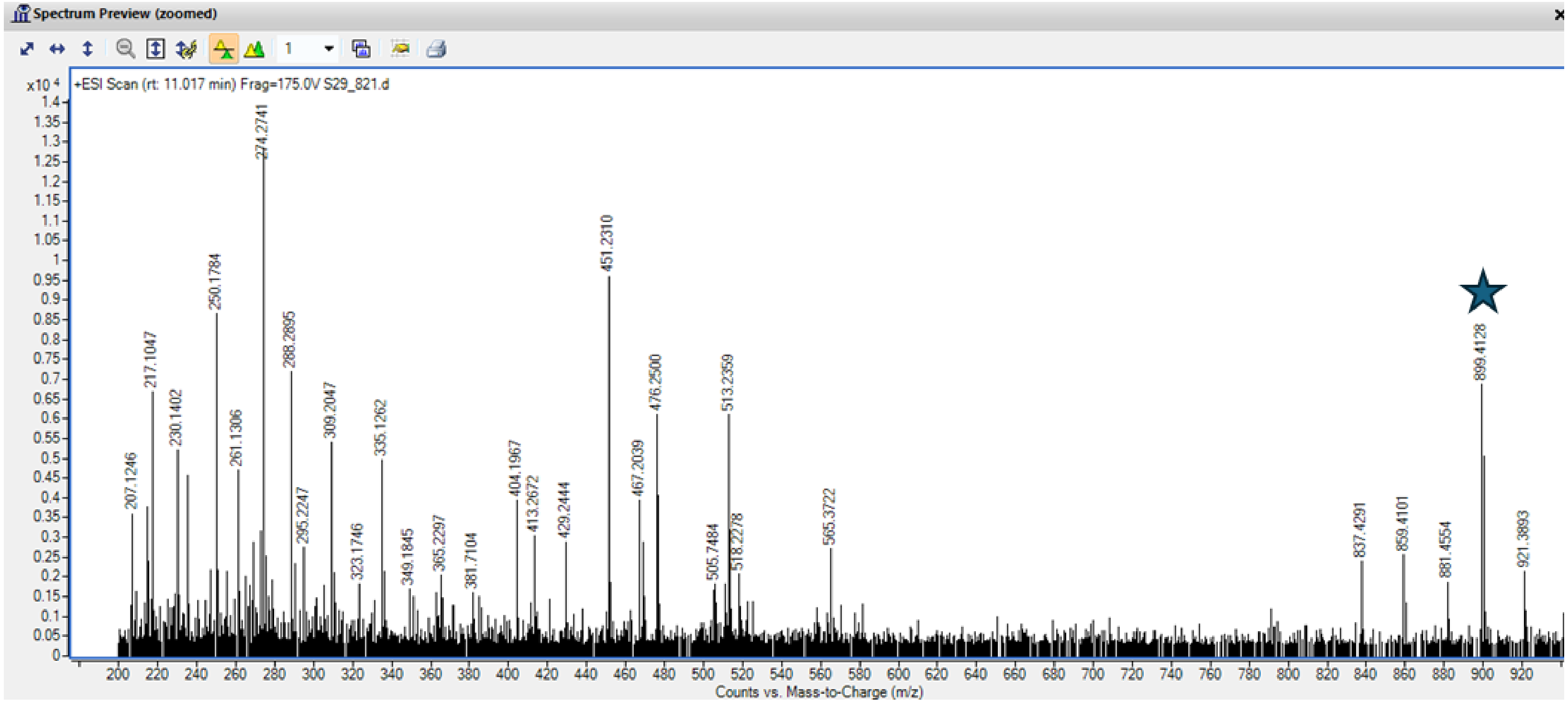

Figure S23. HRMS of ferintoic acid C *m/z* 899.4128 $[M+H]^+$.

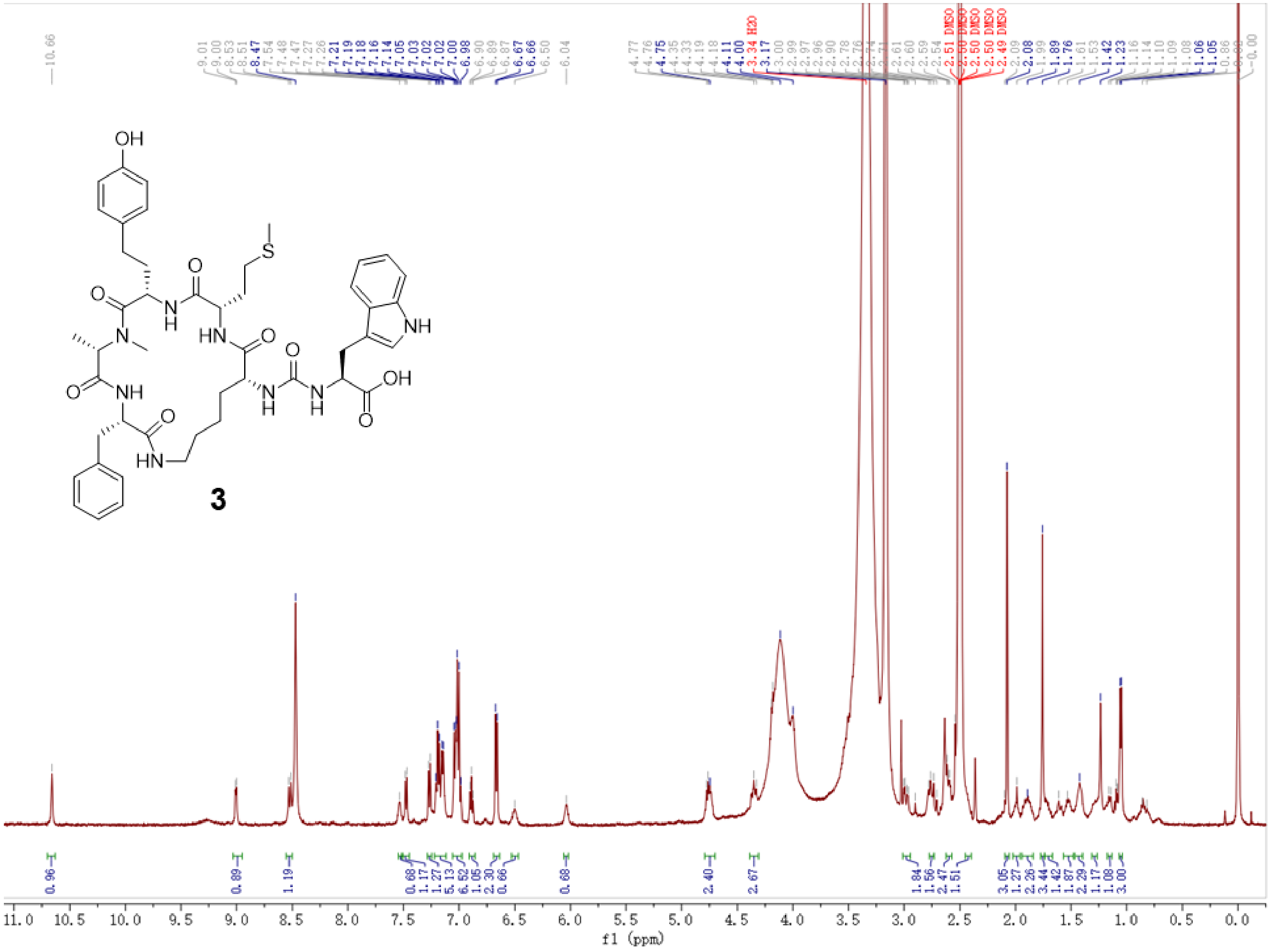

Figure S24. ^{1}H NMR (500 MHz, DMSO-d_6) of ferintoic acid C (**3**).

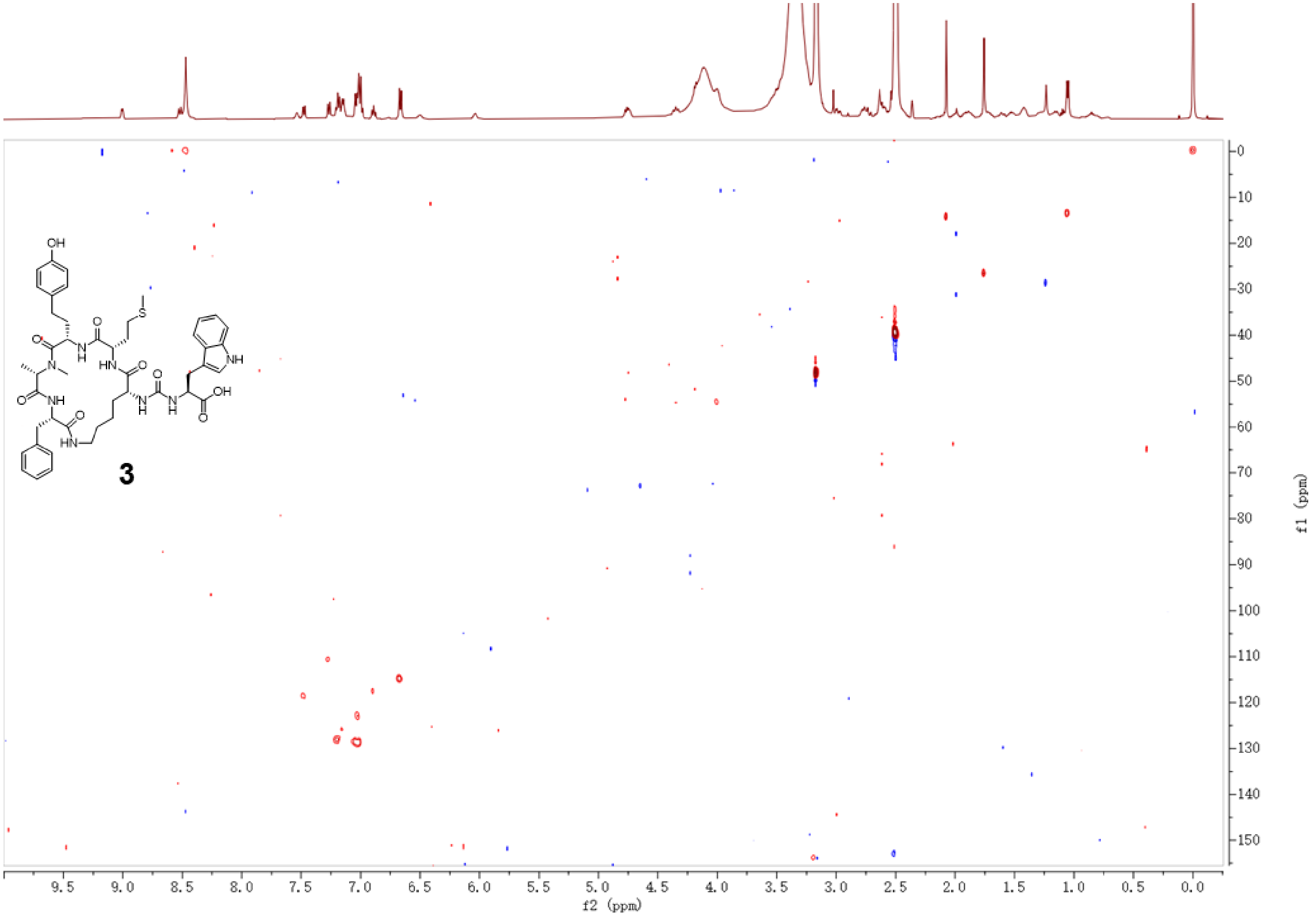

Figure S25. Multiplicity-edited HSQC of ferintoic acid C (**3**).

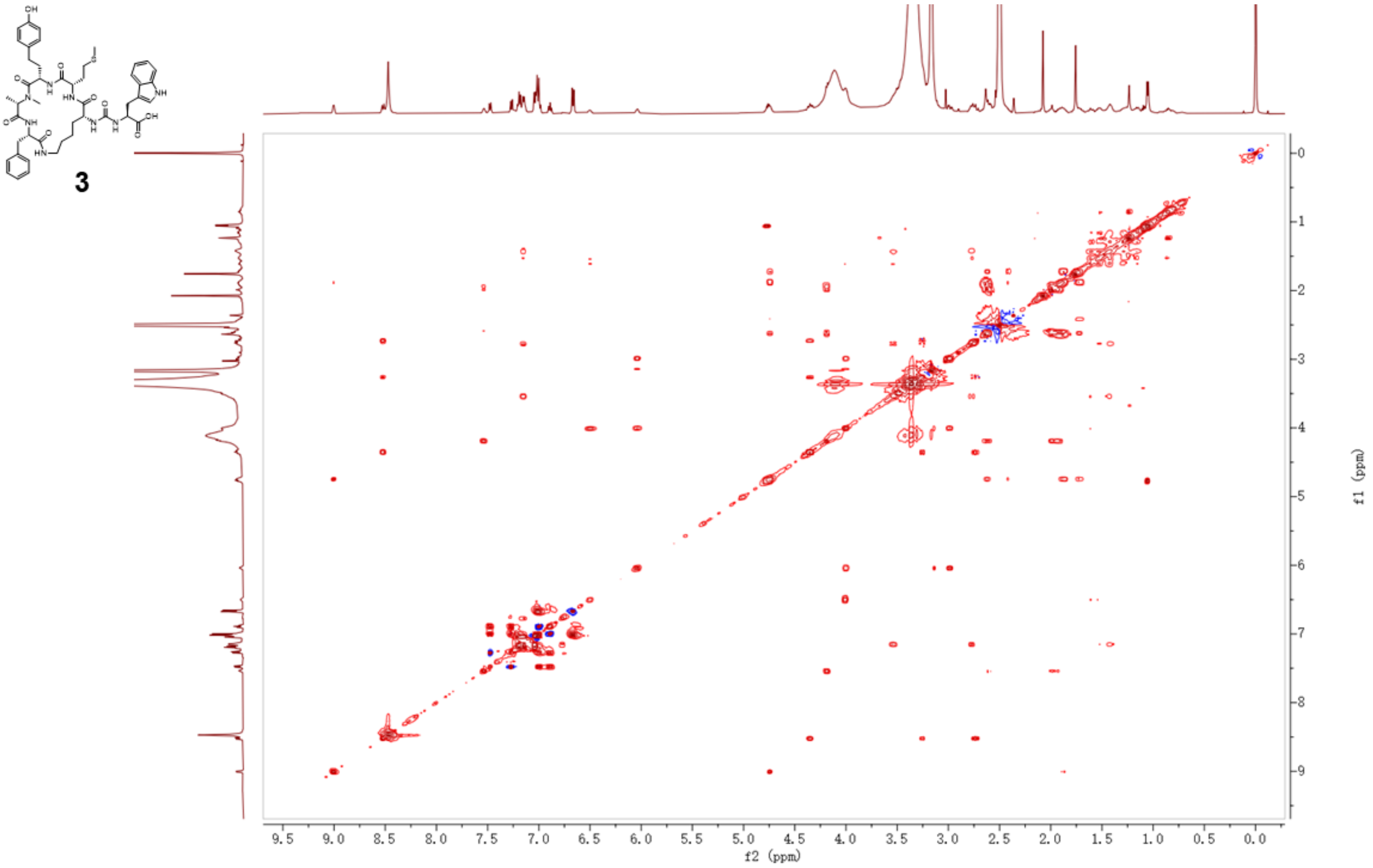

Figure S26. TOCSY of ferintoic acid C (**3**).

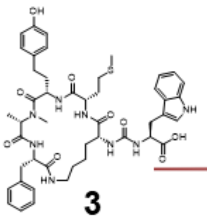

3

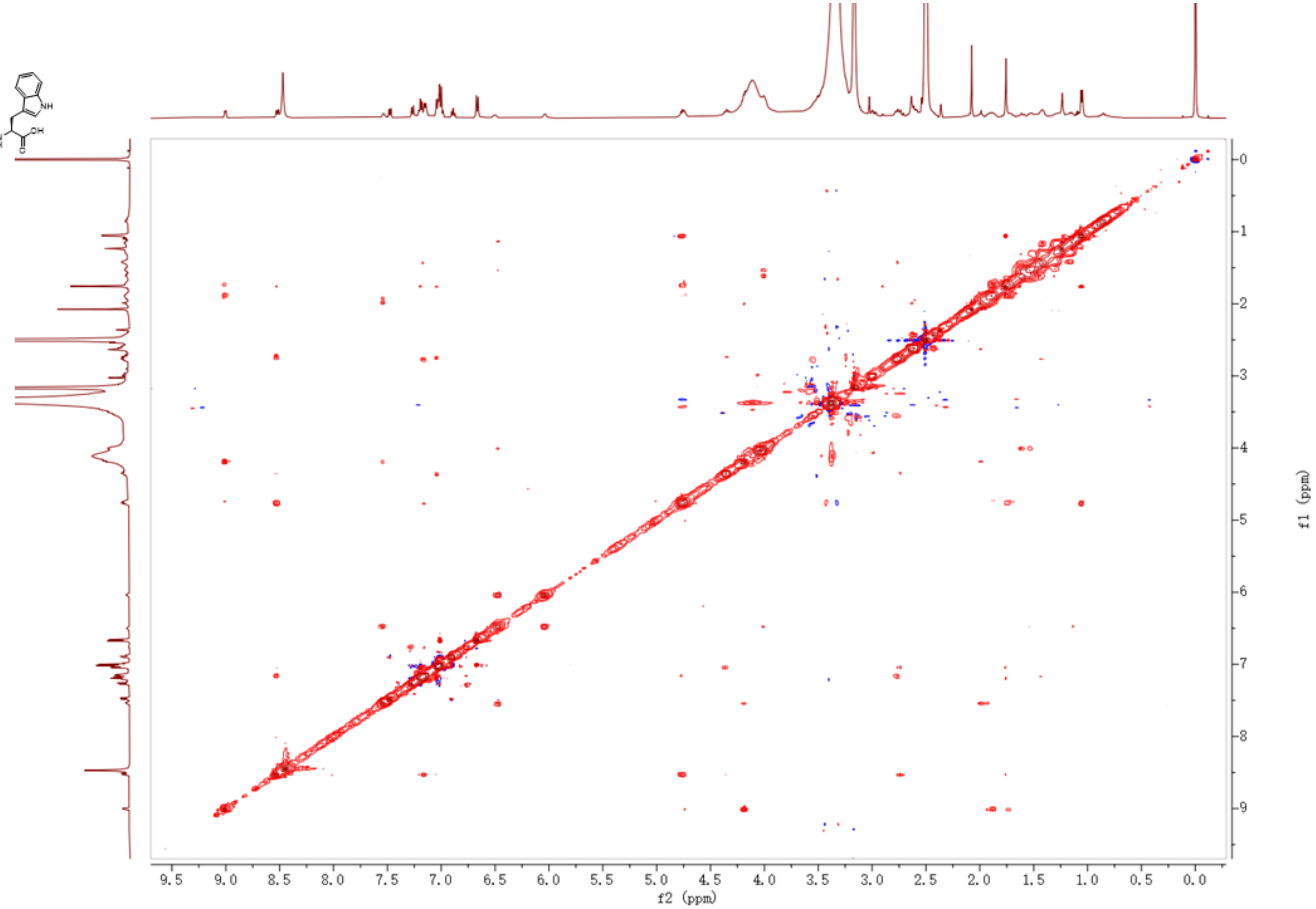

Figure S27. NOESY of ferintoic acid C (**3**).

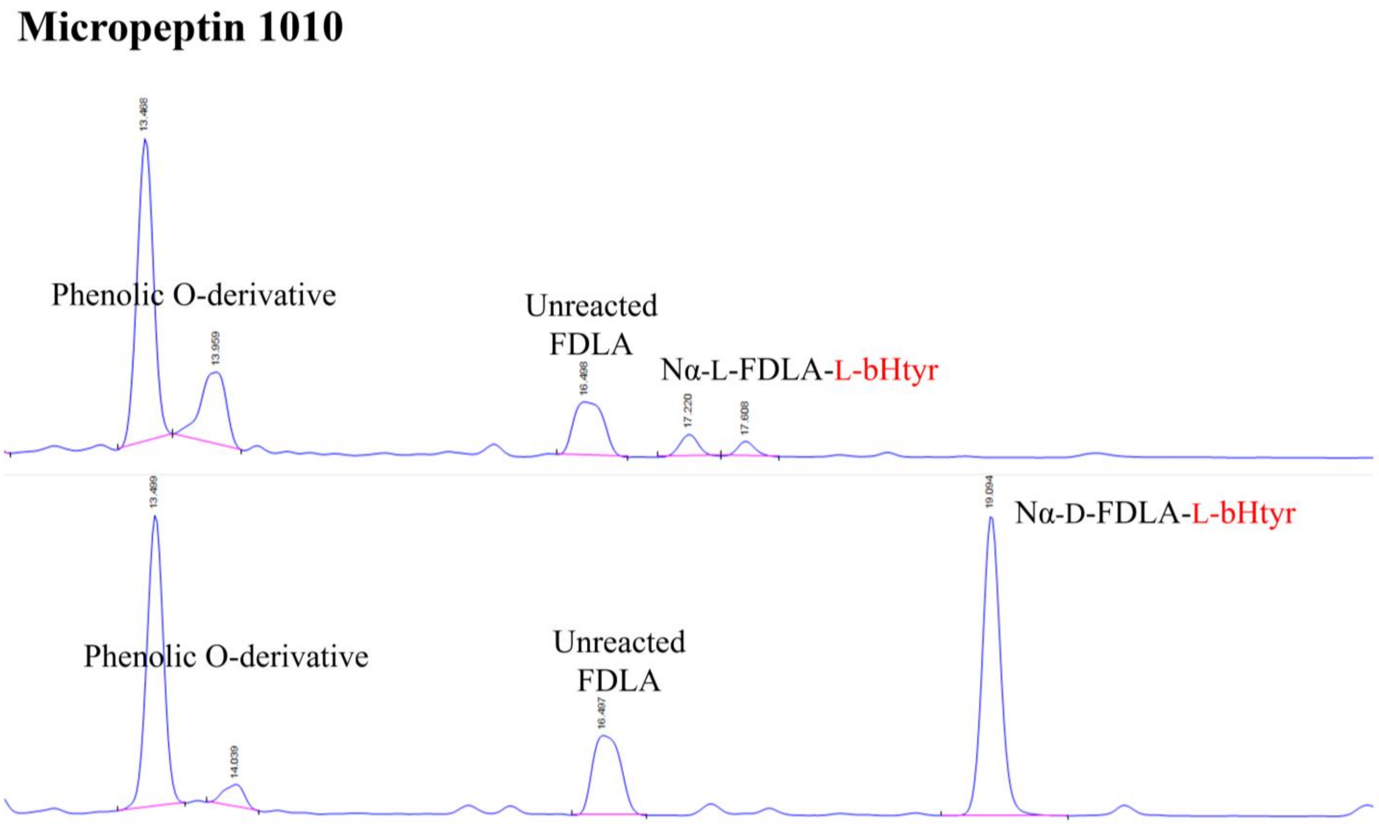

Figure S28. LC-MS analysis of the hydrolysate of **1** reacted with L-FDLA (top panel) and D-FDLA (bottom panel) to determine the configuration of the bHtyr in **1**.

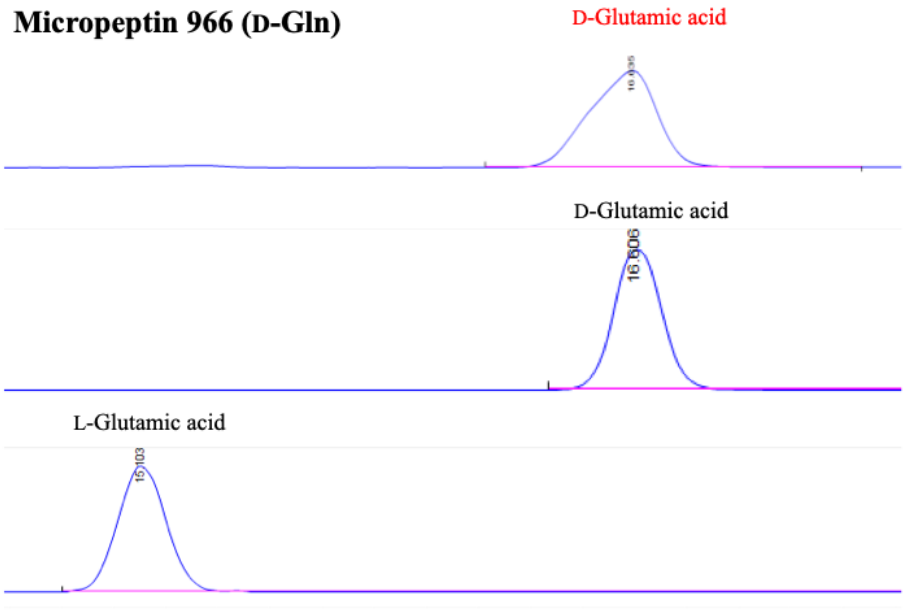

Figure S29. LC-MS analysis of the hydrolysate of **2** (top panel) and L- and D-Glutamic acid (middle and bottom panel, respectively) derivatized with L-FDVA.

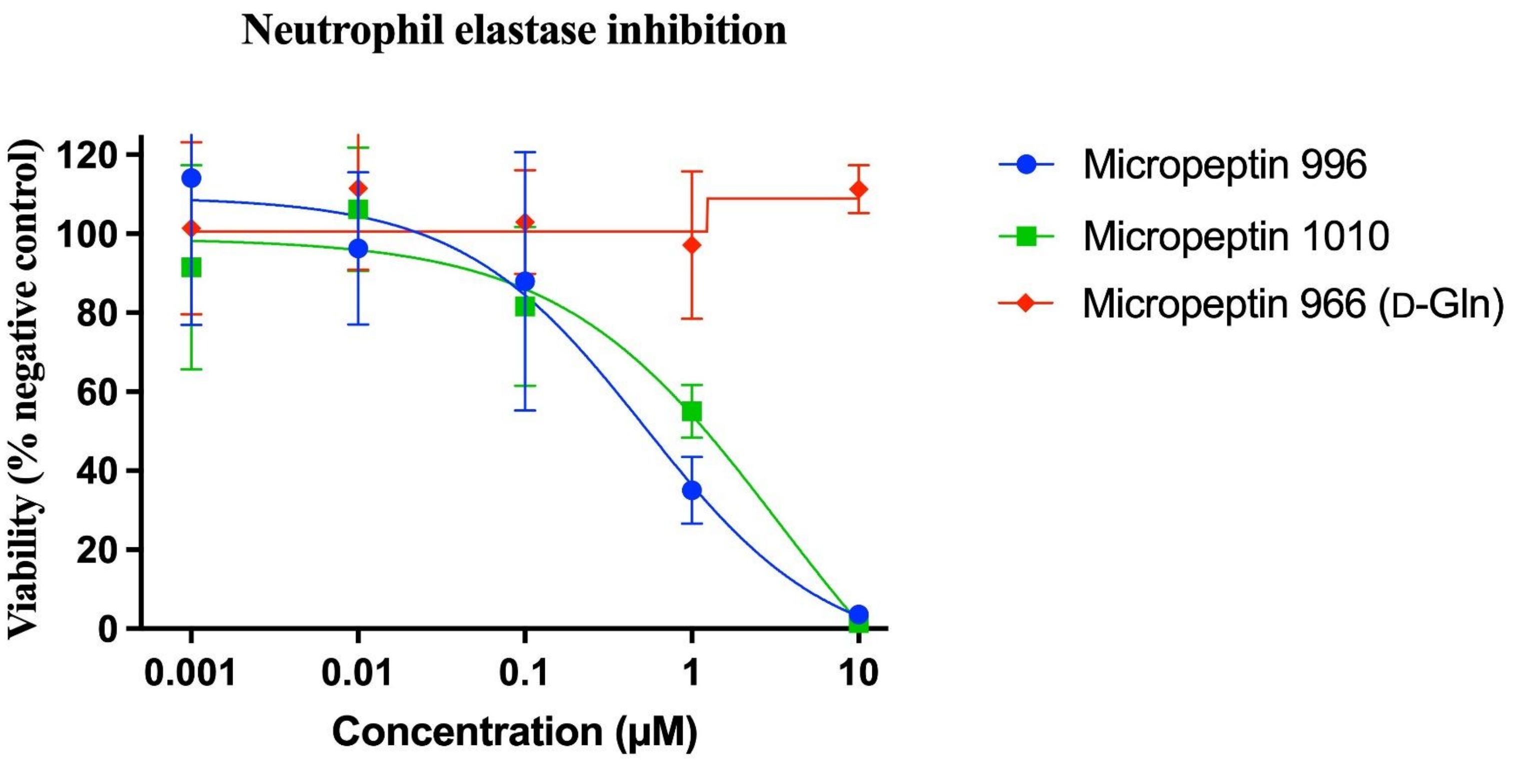

Figure S30. Activity of micropeptin 996 (L-Gln), micropeptin 1010 (**1**), and micropeptin 996 (D-Gln) (**2**) against human neutrophil elastase.

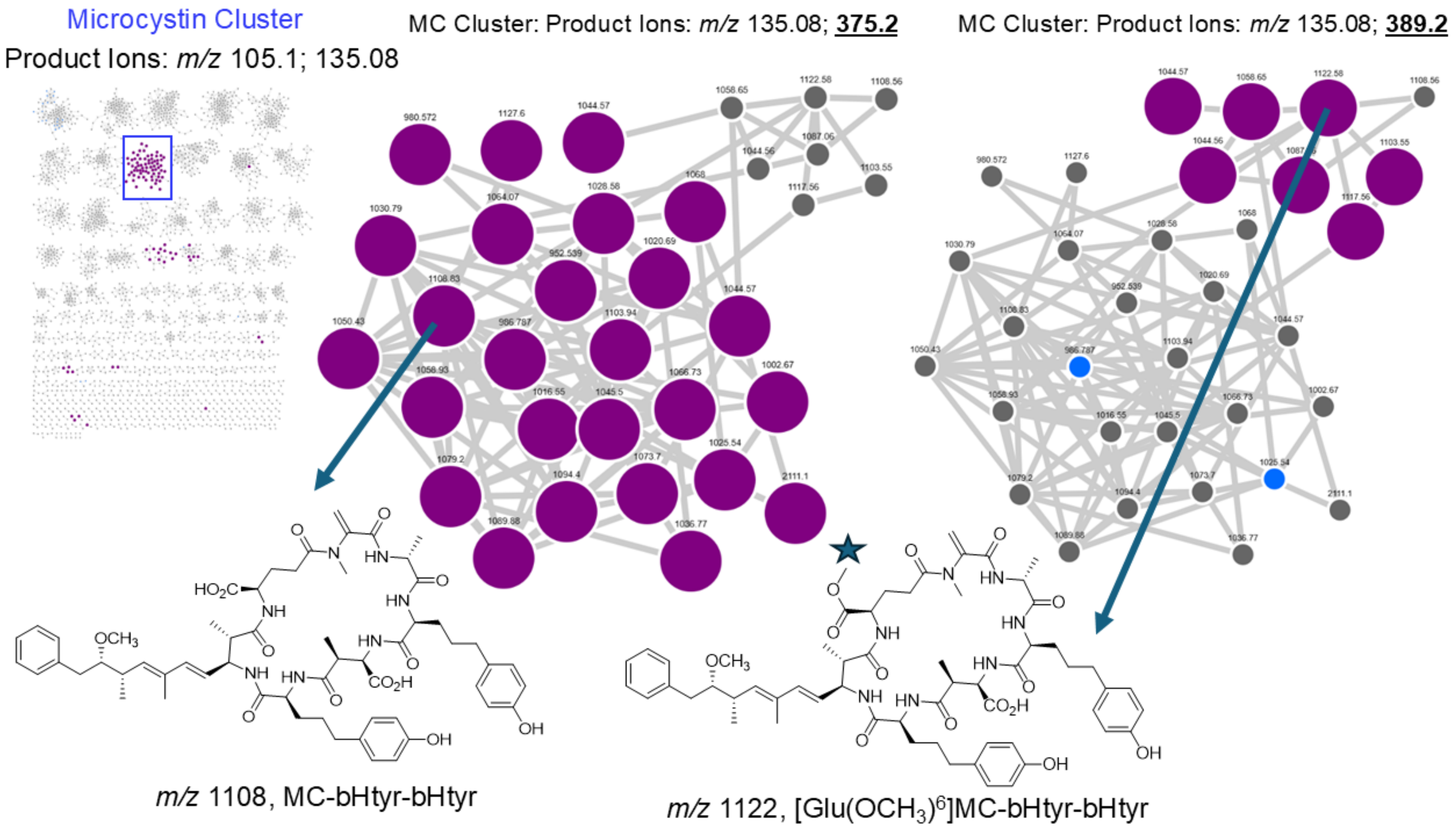

Figure S31. Microcystin cluster in MS/MS molecular network subjected to two different product ion searches: *m/z* 135.08 and *m/z* 375.2 and then *m/z* 135.08 and *m/z* 389.02 to illustrated microcystins with likely [Glu(OCH_3)^6 modifications, which is also supported by the annotation of library microcystins shown.

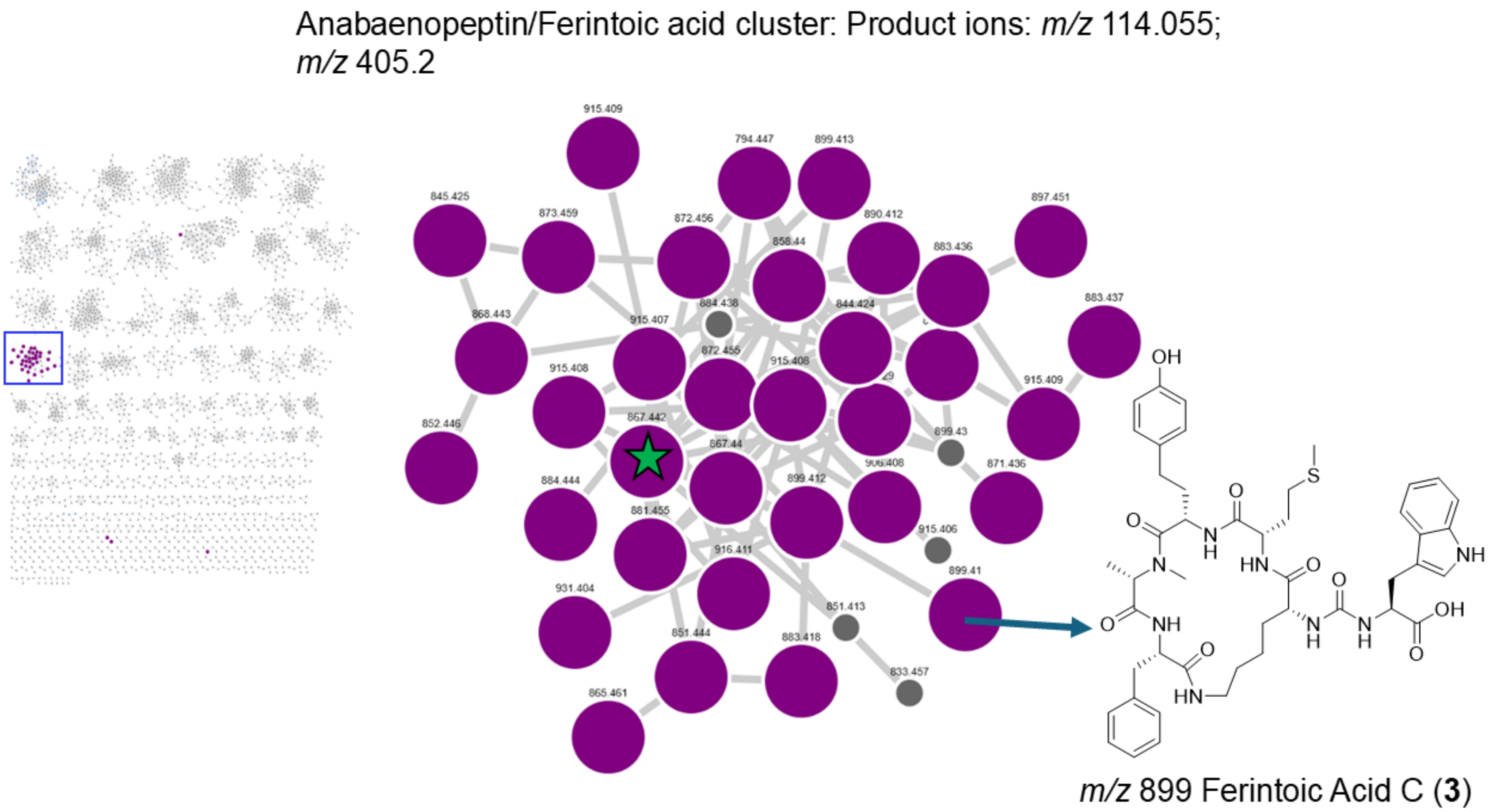

Figure S32. MS/MS cluster of anabaenopeptins/ferintoic acids annotated via product ion searching. Ferintoic acid A (green star) and ferintoic acid C (**3**) were validated using our standard library.

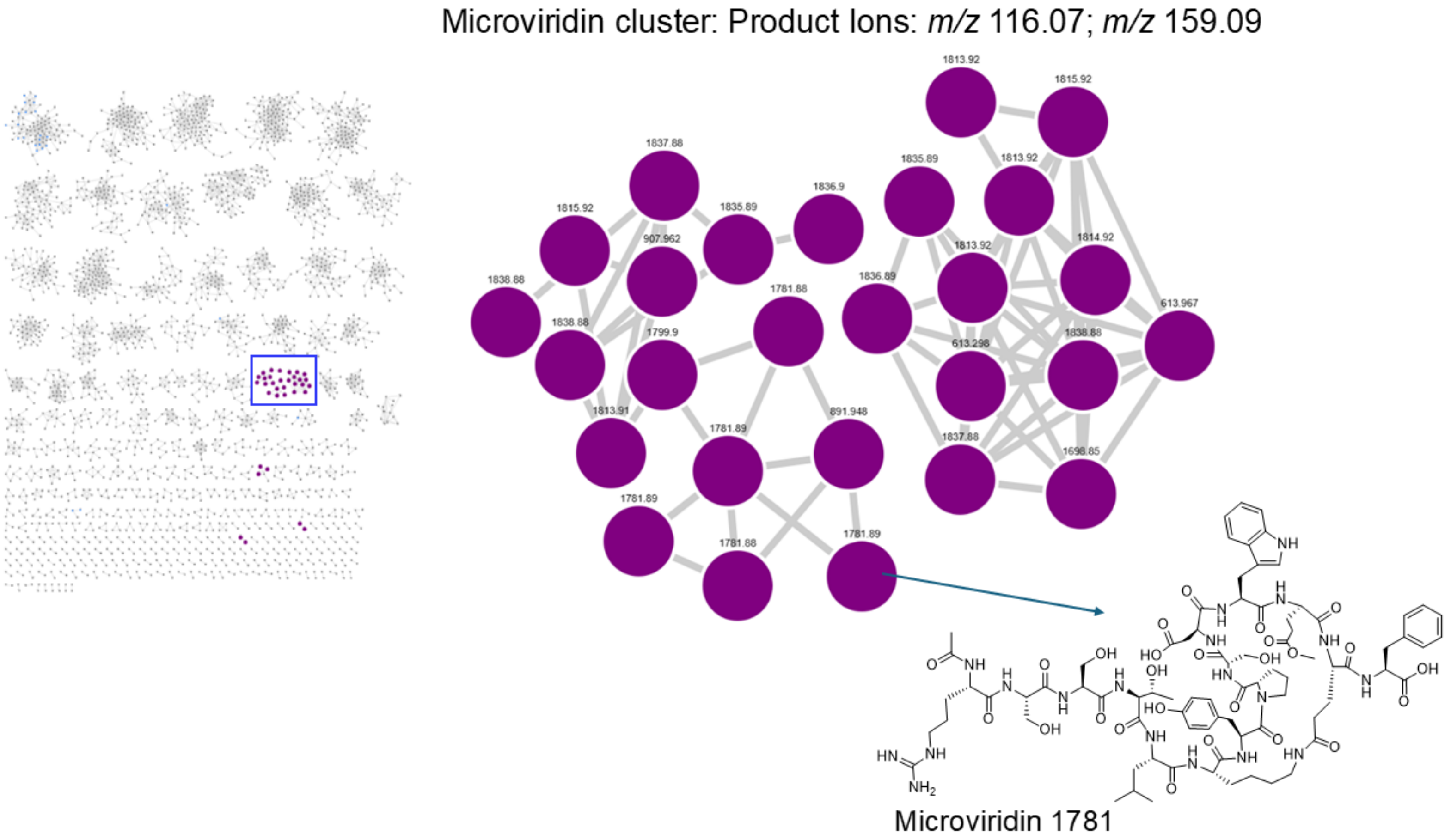

Figure S33. Microviridin cluster annotated using product ion searching in MS/MS network. Standard compound microviridin 1781 was used for validation.

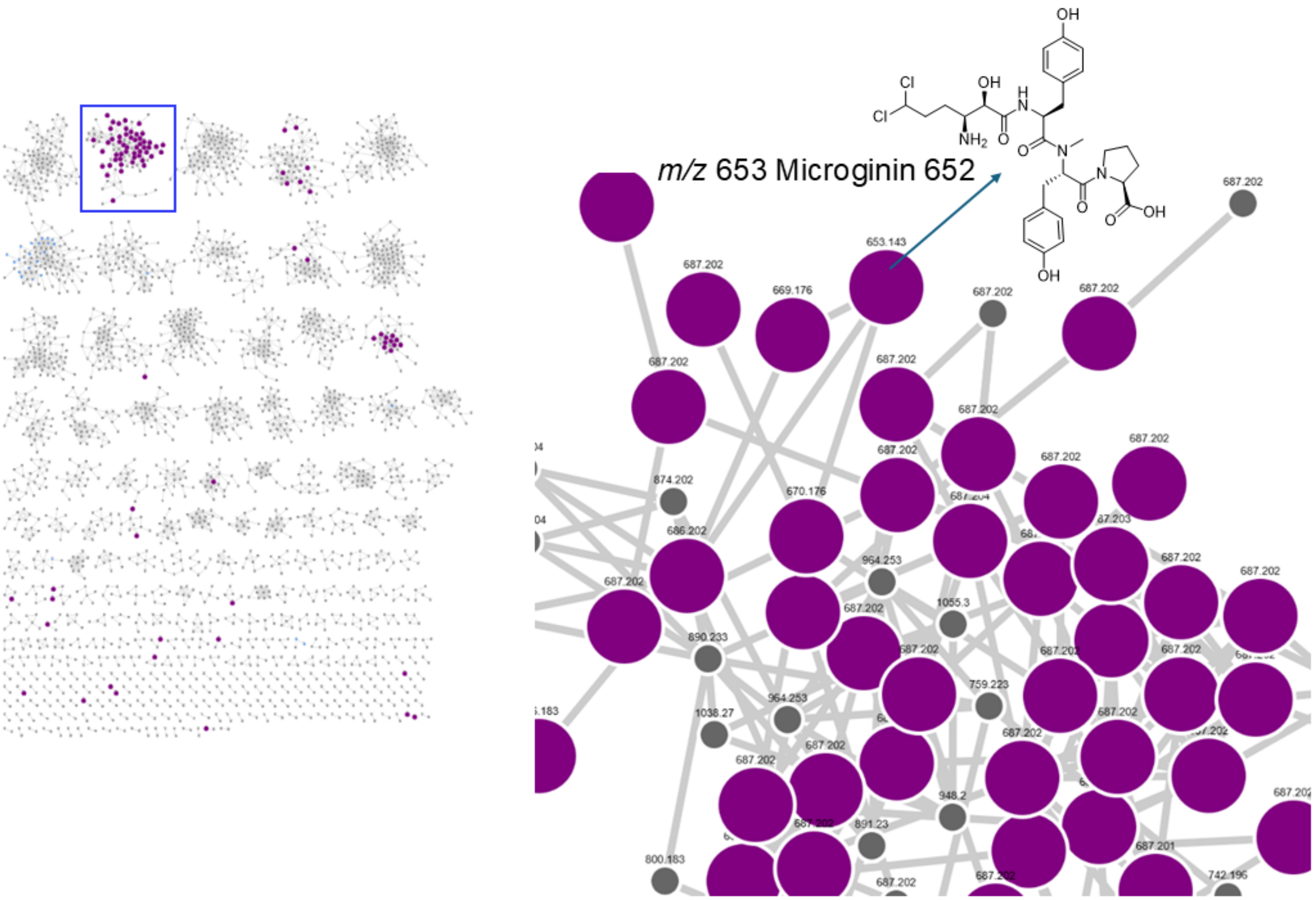

Figure S34. Microginin cluster annotated using product ion searching in MS/MS networks (see main text for product ion search details). Standard compound microginin 652 was used for validation.

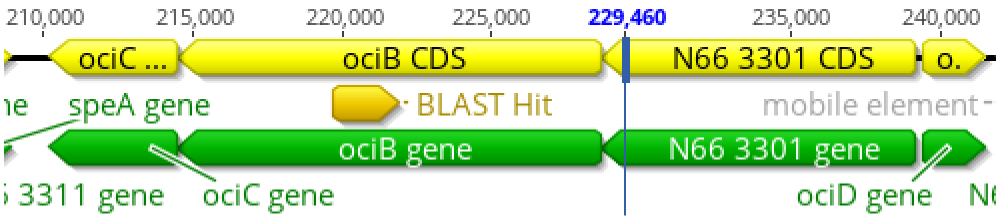

Figure S35. Blast hit to the *ociB* gene (cyanopeptolin biosynthetic pathway) in the metagenomic sequence data from Lake Erie (Miller Road Park).

REFERENCES

1) Fujii, K.; Sivonen, K.; Kashiwagi, T.; Hirayama, K.; Harada, K. -I. Nostophycin, a Novel Cyclic Peptide from the Toxic Cyanobacterium *Nostoc* sp. 152. *J. Org. Chem*. **1999**, *64* (16), 5777–5782.
